# Multi-Platform Analysis of MicroRNA Expression Measurements in RNA from Fresh Frozen and FFPE Tissues

**DOI:** 10.1371/journal.pone.0052517

**Published:** 2013-01-31

**Authors:** Christopher P. Kolbert, Rod M. Feddersen, Fariborz Rakhshan, Diane E. Grill, Gyorgy Simon, Sumit Middha, Jin Sung Jang, Vernadette Simon, Debra A. Schultz, Michael Zschunke, Wilma Lingle, Jennifer M. Carr, E. Aubrey Thompson, Ann L. Oberg, Bruce W. Eckloff, Eric D. Wieben, Peter Li, Ping Yang, Jin Jen

**Affiliations:** 1 Medical Genome Facility Gene Expression Core, Mayo Clinic, Rochester, Minnesota, United States of America; 2 Department of Health Sciences Research, Division of Biomedical Statistics & Informatics, Mayo Clinic, Rochester, Minnesota, United States of America; 3 Experimental Pathology, Mayo Clinic, Rochester, Minnesota, United States of America; 4 Cancer Basic Science Research, Cancer Biology, Mayo Clinic, Jacksonville, Florida, United States of America; 5 Medical Genome Facility Sequencing Core, Mayo Clinic, Rochester, Minnesota, United States of America; 6 Department of Health Sciences Research, Division of Epidemiology, Mayo Clinic, Rochester, Minnesota, United States of America; University of Connecticut Health Center, United States of America

## Abstract

MicroRNAs play a role in regulating diverse biological processes and have considerable utility as molecular markers for diagnosis and monitoring of human disease. Several technologies are available commercially for measuring microRNA expression. However, cross-platform comparisons do not necessarily correlate well, making it difficult to determine which platform most closely represents the true microRNA expression level in a tissue. To address this issue, we have analyzed RNA derived from cell lines, as well as fresh frozen and formalin-fixed paraffin embedded tissues, using Affymetrix, Agilent, and Illumina microRNA arrays, NanoString counting, and Illumina Next Generation Sequencing. We compared the performance within- and between the different platforms, and then verified these results with those of quantitative PCR data. Our results demonstrate that the within-platform reproducibility for each method is consistently high and although the gene expression profiles from each platform show unique traits, comparison of genes that were commonly detectable showed that detection of microRNA transcripts was similar across multiple platforms.

## Introduction

Since they were first described, microRNAs (miRNAs) have been studied widely for their role in the regulation of gene expression [1], [2], [3], [4], [5]. MiRNAs are best known for the ability to down-regulate protein expression by directly or indirectly inhibiting transcription or by degrading mRNA transcripts [1], [4], [5], [6], [7], [8]. But they can also activate translation under certain environmental conditions [5]. MiRNAs are usually transcribed from intergenic regions or the antisense strands of genes [9], [10]. However, significant numbers of miRNAs have been discovered in introns and even exons of protein encoding genes [10]. Precursor miRNAs undergo extensive enzyme-mediated processing which results in a single-stranded molecule that is approximately 22 nucleotides in length. In the human genome, more than 1,500 mature miRNA transcripts have been characterized thus far [11].

Functionally, miRNAs can target mRNA molecules involved in many biological processes, including cell growth and development, cell fate, and apoptosis [12], [13], [14]. Given that miRNA transcripts affect nearly every aspect of cellular function, it is not surprising that they play a critical role in the etiology of a wide variety of disease manifestations [15]. Indeed, miRNAs have been implicated in many types of cancers, as well as specific cardiac and neurologic diseases [16], [17], [18], [19], [20], [21], [22], [23]. Furthermore, studies have identified tissue-specific miRNA signatures that have the potential to act as diagnostic markers in human disease [19], [24], [25]. For this reason, it is critical that methods for detection and quantification of miRNAs in a clinical setting are sufficiently sensitive and specific in order to distinguish healthy and disease states.

Research studies have characterized several different platforms for miRNA expression profiling by assaying synthetic RNA or RNA from commercially available cell lines and tissues [26], [27], [28], [29]. Others have described the detection and quantification of miRNA transcripts in samples from both fresh frozen (FF) and formalin-fixed paraffin-embedded (FFPE) tissues from human patients [30], [31]. These studies have highlighted the great diversity of methods that are available for miRNA expression analysis. Notably, these technologies exhibit different dynamic ranges and resolution capabilities, making it difficult to determine true miRNA expression levels.

Gene expression microarrays are relatively inexpensive and are useful for profiling the miRNA transcriptome in a single experiment. However, studies have shown significant variability between different microarray platforms for miRNA profiling [26], [28]. The evolution of digital counting techniques provides a new way to profile miRNA expression. NanoString technology employs unique fluorescent–tagging of individual miRNA species followed by two-dimensional display and optical scanning and counting of miRNA molecules [32]. More recently, advances in Next Generation Sequencing (NGS) have enabled a comprehensive evaluation of the miRNA transcriptome that allows for the characterization of novel transcripts [33]. Although the cost of NGS technology is decreasing, it remains prohibitive for many laboratories, and data analysis pipelines are still maturing. Therefore, researchers continue to use microarrays and other hybridization-based technologies to measure miRNA expression, prompting questions about how data from these platforms can be compared.

In this study, we compared Affymetrix, Agilent, and Illumina microarray platforms with each other and with NanoString miRNA counting and NGS miRNA-Seq technologies by analyzing miRNA expression in total RNA samples from FF and FFPE lung tissues as well as a lung cancer cell line. A subset of these data was also compared to real-time PCR data generated from the same samples by using the Fluidigm BioMark System.

## Results

### Performance of miRNA Expression Profiling Analysis within Each Platform

**Figure 1:**
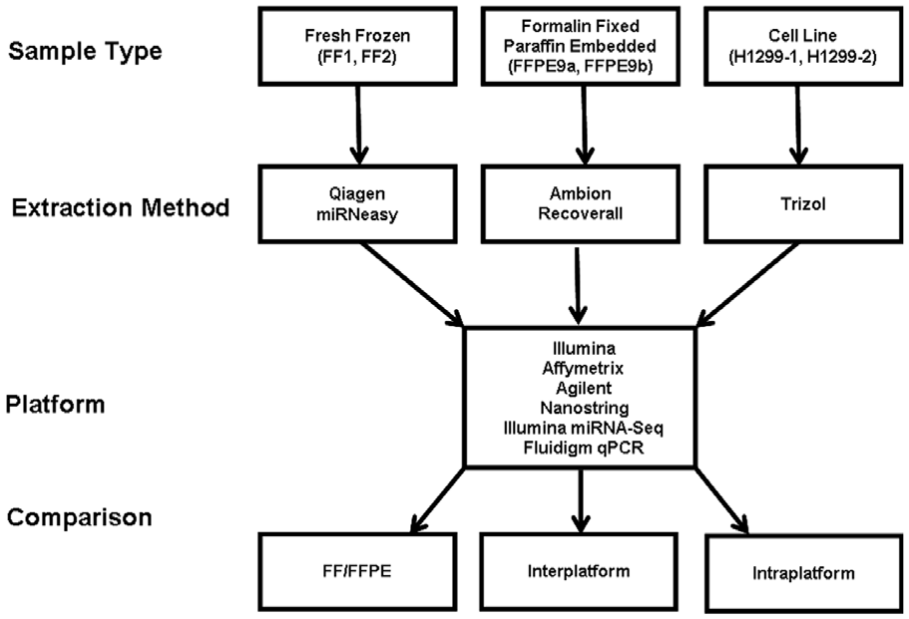
Experimental design of the miRNA expression platform comparison. RNA from replicate samples derived from normal lung, lung tumor, and a cell line were extracted by methods as indicated. All samples were subsequently analyzed by Illumina, Affymetrix, Agilent, NanoString, Illumina miRNA-Seq, and Fluidigm qPCR.

Total RNA extracted from lung samples FF1, FF2, FFPE9a, FFPE9b, and cell line H1299 was used as input material for intra- and inter- platform comparisons of miRNA expression assays (Figure 1). MiRNA detection counts varied according to the sample type and miRNA expression platform (Table 1). For the Affymetrix microarray platform, the number of detected transcripts ranged from 340 for FF2 to 221 for H1299-2. Intra-platform Pearson correlations (r) of the replicates ranged from 0.951 to 0.974. Agilent results for FF and FFPE were relatively consistent across the two tissue sample types. However, H1299-1 and H1299-2 lung cancer cell lines demonstrated lower detection counts, with 74 and 87 miRNA transcripts, respectively. The number of detected genes for the Illumina microarray platform ranged from 482 in sample FF2 to 562 miRNA transcripts in the H1299 cell line. Replicate correlations for this platform ranged from 0.932 for FFPE samples to 0.985 for the FF samples. The miRNA detection count obtained by the NanoString platform ranged from 350 for FF2 to 76 for H1299-1 and replicate correlations ranged from 0.643 to 0.989. MiRNA-Seq detection counts ranged from 650 for FFPE9a to 472 for H1299-1. Replicate correlations ranged from 0.916 for H1299 to 0.935 for FFPE9 samples.

**Table 1:** Replicate performance of tested miRNA platforms.

	Affymetrix* (n = 847)		Agilent (n = 719)		Illumina (n = 858)		NanoString (n = 654)		NGS (n = 792)	
Sample	Detected Transcripts	r	Detected Transcripts	r	Detected Transcripts	r	Detected Transcripts	r	Detected Transcripts	r
FF1	249	0.974	266	0.985	498	0.985	257	0.958	569	0.934
FF2	340		256		482		350		510	
FFPE9a	295	0.970	227	0.936	508	0.932	250	0.989	650	0.935
FFPE9b	329		223		495		270		585	
H1299-1	249	0.951	74	0.992	536	0.984	76		472	0.916
H1299-2	221		87		562		86	0.643	521	

* The miRNA transcripts interrogated by each platform were assessed based on platform-specific metrics. n =  number of interrogated transcripts by each platform and were used to calculate the Pearson Correlations(r).

### Reproducibility of miRNA Profiling between FF and FFPE Samples

**Figure 2:**
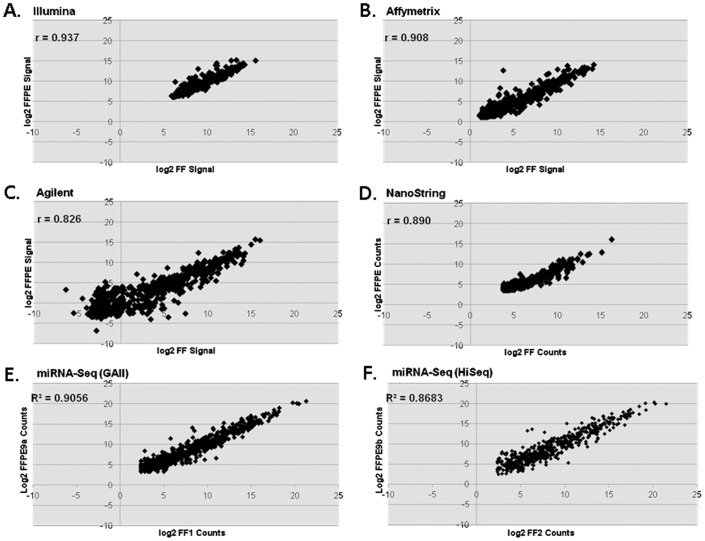
Expression correlations of data derived from fresh frozen (FF) and paraffin-embedded (FFPE) samples. Correlations of log2 transformed signal counts for each platform are shown (A–F) along with the respective Pearson correlation (r) coefficients. The average expression values of two replicates were used except for miRNA-Seq, where individual samples were directly compared as indicated.

We further assessed the performance of each platform by comparing expression values obtained from matched FF and FFPE samples (Figure 2). The overall tissue type did not appear to significantly affect the miRNA profiling and the correlation across sample types ranged from r = 0.826 for the Agilent microarray platform to 0.937 for the Illumina microarray. For miRNA-Seq analysis, the two replicates were analyzed using two different Illumina sequencers (GAII vs. HiSeq2000) and they gave similar correlations, with r = 0.906 and 0.868, respectively. The expression range of the data, as measured by log_10_ signal intensity, was the greatest for miRNAseq (5.4 log), followed by Agilent (4.8 log), Affymetrix (4.0 log), NanoString (3.7 log), and Illumina (2.7 log).

### Cross-platform Comparisons

Among the miRNA targets, we identified 484 transcripts that were commonly interrogated among all tested platforms and we used this set for cross-platform comparisons (Figure S1). For FF and its matched FFPE sample, the number of detected miRNA transcripts was similar for the Affymetrix, Agilent, and NanoString platforms, but varied considerably for Illumina and miRNA-Seq. For sample FF1, detection of commonly interrogated miRNA ranged from 35.33% for Affymetrix to 69.42% for miRNA-Seq (Table S1). As expected, sample FF2 gave similar results. However, detection by Affymetrix and NanoString was nearly 10% higher in FF2 than FF1. FFPE samples gave nearly identical detection rates, ranging from 32% by Agilent to greater than 70% for miRNA-Seq. Cell line H1299 samples also demonstrated a similar level of detection within each platform. However, the number of detected miRNA transcripts in H1299 were, overall, lower than for the fresh frozen or FFPE samples. Indeed, both Agilent and NanoString platforms exhibited detection calls only 12% to 14% of the commonly interrogated transcripts in H1299 cells. In contrast, Illumina-detected miRNA were nearly five-fold higher than the other platforms in H1299 cells.

To assess the agreement of miRNA transcript detection across platforms, as well as the criteria used by each platform to determine detected/present calls, we used the 484 commonly interrogated transcripts to make platform-to-platform comparisons for each sample (Figure S2). The number of detected transcripts for Affymetrix, Agilent, and NanoString platforms was similar within a sample. Across samples, the number of detected transcripts was also relatively consistent for these platforms, with the exception that fewer miRNA were detected in the cell lines H1299-1 and H1299-2 (Table S2). The Illumina and miRNA-Seq comparison showed that these platforms detected transcripts similarly across the sample types. Some of the miRNA transcripts were almost universally expressed in all tested samples and detected at relatively consistent levels across all platforms (Table S4 A–F). Examples of these miRNAs are miR-26a, let-7a, and miR-24. Transcripts let-7b and miR-23a were present in the top 50 ranked genes in FF and FFPE samples across all platforms. But they did not appear in this ranking among the H1299 cell line replicates.

### MicroRNA Expression Patterns in Tested Lung Tissues

**Figure 3:**
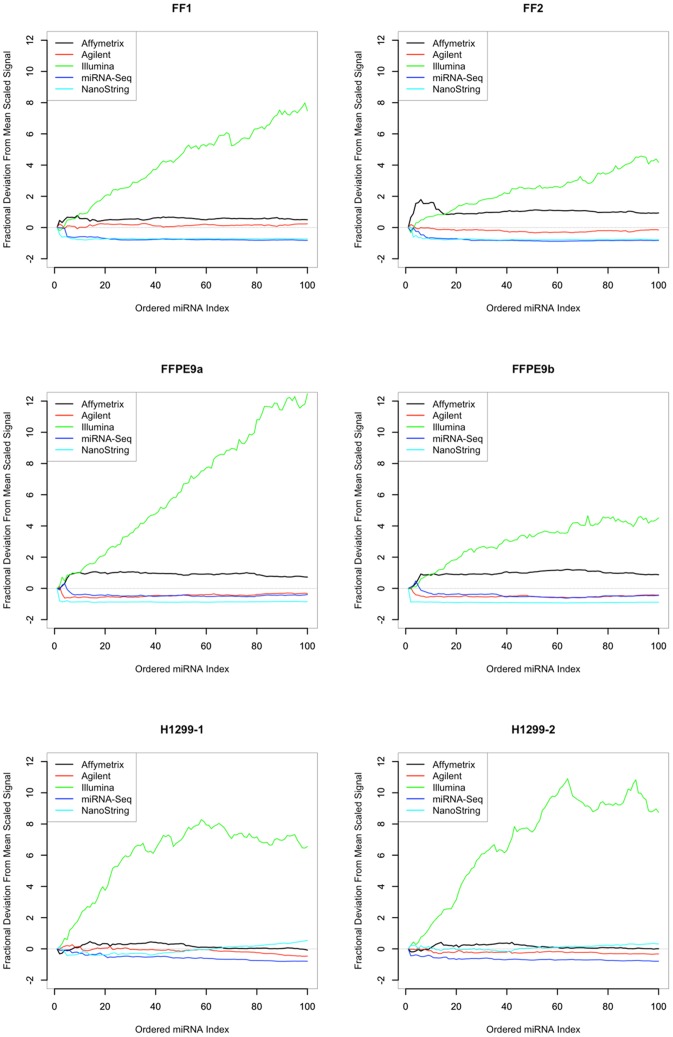
Fractional deviation from the mean miRNA expression for the top ranked 100 miRNA transcripts. For each sample (A–F), the fractional deviation was plotted by each platform against the mean scaled expression of the ranked miRNA transcripts.

Next, we assessed the overall distribution of miRNA expression by plotting the fractional deviation of the mean scaled signal intensity for the top 100 miRNA transcripts in each sample across each of the miRNA platforms (Figure 3). The distribution of expression values across all platforms was relatively consistent, although the ranked order of specific miRNA transcripts differed among the platforms for the same sample (Table S4 A–F). Interestingly, Affymetrix, Agilent, miRNA-Seq, and NanoString demonstrated similar patterns of signal across each sample type. However, the Illumina platform was clearly an outlier in this analysis, exhibiting the highest overall percent maximum signal.

### Comparison to Quantitative PCR by Fluidigm Dynamic Array

We compared the expression fold changes between FF1/H1299-1 and FFPE9a/H1299-1 with miRNA expression differences obtained by RT-PCR using the Fluidigm dynamic array (South San Francisco, CA) and ABI Taqman miRNA assays (Foster City, CA; Table 2). We used Fluidigm-based qPCR to study 41 miRNAs that were shared in the FF1 sample across all miRNA platforms.

**Table 2:** Correlation of miRNA expression fold-change in Fluidigm-based qPCR compared to five other miRNA gene profiling platforms.

FF1						FFPE 9a				
	Affymetrix	Agilent	Illumina	NanoString	miRNA-Seq	Affymetrix	Agilent	Illumina	NanoString	miRNA- Seq
r_s_	0.6308	0.4937	0.5113	0.5932	0.7045	0.4611	0.3516	0.3350	0.4808	0.4720
p-value	<0.0001	0.0010	0.0006	<0.0001	<0.0001	0.0041	0.0329	0.0427	0.0026	0.0032
n	41	41	41	41	41	37	37	37	37	37

* The number of miRNA transcripts shared among all platforms and detected by qPCR in the fresh frozen and formalin-fixed paraffin embedded samples as indicated. Spearman correlation coefficient (r_s_) and its associated p-value are indicated.

The miRNA-Seq platform demonstrated the highest correlation with Fluidigm qPCR for RNA isolated from FF tissues (r = 0.7045, p<0.0001), while its correlation with Affymetrix, NanoString, Illumina, and Agilent were respectively lower but still statistically significant (p<0.001). For FFPE sample, 37 transcripts were shared and assessed by quantitative PCR. NanoString demonstrated the highest correlation (r = 0.4808, p = 0.0026). The miRNA-Seq platform demonstrated the second best FFPE sample correlation with the qPCR data (r = 0.4720, p = 0.0032), followed by Affymetrix, Agilent, and Illumina. For the qPCR data derived from the FF1 sample, six miRNA transcripts (miR-16, miR-27a, miR20a, let-7f, mir96, and miR-29b) gave log ratio values that were disparately lower than log ratios derived by the Affymetrix, Agilent, Illumina, and Nanodrop platforms (Table S3a). However, log ratios derived by miRNA-Seq were consistent with that of qPCR for all six of these transcripts. As reflected by the lower overall correlation values (Table 2), the relative expression of the FFPE9a sample indicated that qPCR-based expression was highly divergent in nine of 37 miRNA transcripts with the other expression platforms (let-7a, miR-125a-5p, miR-31, miR-484, miR-16, miR-455-3p, miR-26b, let-7f, and miR-29b; Table S3b).

## Discussion

Herein we performed an extensive comparison of five different miRNA expression profiling platforms using total RNA from tissue-matched fresh frozen and FFPE samples. Our results demonstrate that all platforms perform consistently in replicate runs for all sample types. We also demonstrated that within each platform, miRNA profiling of RNA from matched fresh frozen and formalin-fixed paraffin-embedded samples is highly reproducible and strongly correlated. Affymetrix, Agilent, and NanoString platforms gave detection calls that were similar to each other, despite each having a different number of transcripts available for detection. The number of detected transcripts for Illumina and miRNA-Seq was substantially higher than the other platforms and similar to each other. Because of its quantitative nature, the expression range was significantly wider for miRNA-Seq, followed by Agilent and Affymetrix arrays. In our hands, the Illumina array provided the smallest expression range among the different platforms (Figure 2). This may reflect a systematic effect that results from the Illumina labeling technique and subsequent PCR-based amplification.

We also considered the capacity of an individual platform to detect miRNA among the 484 genes commonly recognized by all platforms that were tested. Using this approach, the similarity of Affymetrix, Agilent, and NanoString detection calls in the FF and FFPE sample types remained, as did that of Illumina and miRNA-Seq (Figure S1). This may reflect the differences among the individual technologies as well as the detection algorithm for each platform. The Illumina assay was a PCR-based assay that incorporated 34 amplification cycles, while the other array assays are based primarily on non-amplified templates that hybridize to complementary sequences present in the array or assay system. For this reason, the miRNA expression signal for the Illumina platform deviated significantly from the mean, appearing as an outlier from the other expression platforms.

Tumor cell lines, as monoclonal expansions of a relatively homogenous cell population, are generally regarded to express a more restricted miRNA profile as compared to multi cell type tissue samples [34]. Consistent with this notion, we observed that the average number of detected miRNA genes were lower for four of the five tested platforms, despite the fact that different labeling strategies and detection algorithms were utilized. The exception in this case was the PCR-based Illumina system.

Because the true number of miRNA expressed within a tissue is unknown and this value is subject to the method used for miRNA detection as well as the detection parameters of the platform, we assessed the level of agreement by pairwise platform comparisons. Across all sample types, Illumina and miRNA-Seq gave the highest average level of agreement among the commonly detected transcripts. This level of agreement is likely due in part to the fact that these two platforms detect the most miRNAs, through PCR amplification of the templates and digital sequencing, respectively. Illumina incorporated a 34 cycle amplification, whereas the mRNA-seq assay used 12 cycles. However, Illumina was clearly an outlier in this analysis, suggesting that assay-specific factors were involved.

Pairwise comparison of Affymetrix/Illumina and Affymetrix/miRNA-Seq also demonstrated agreement for all but the FF1 sample (Figure S2), suggesting that the lower detection calls for this sample may have contributed to lower inter-platform concordance. Additionally, we compared the expression values obtained by each of these five platforms with those obtained by qPCR using 41 (FF) and 37 (FFPE) shared miRNA transcripts. We found that for FF samples, the miRNA-Seq platform exhibited the highest correlation with the qPCR assay (Table 2), followed closely by the Affymetrix platform. Though the FF correlations were relatively low, they were significantly higher than those of the FFPE comparison. However, the apparent low overall correlation between each tested platform and qPCR could also be affected by the specificity and robustness of the qPCR assays. In this regard it is interesting to note that recent evidence indicates wide spread editing of miRNA molecules, even within the seed region, that may have affected the target of the ABI miRNA qPCR assays employed in this study [35]. The absence of a method to accurately measure the true miRNA expression in a given sample continues to make cross platform comparative studies such as this difficult.

Indeed, others have compared miRNA expression profiling methods, although their platform assessments were not as comprehensive as was the current study [26], [28], [29], [36]. These studies also found substantial inter-platform differences. However, our analysis of transcripts that were commonly interrogated demonstrated general similarities in the level of expression across platforms. Particularly for the most abundantly expression miRNA genes, we observed that a significant fraction were consistently detected by all or most of the tested platforms (Table S4 A–F).

Therefore, with few exceptions, the choice of platform for miRNA expression profiling will be heavily dependent upon the primary objective of the study. If the purpose of the study is to determine the relative expression of miRNA genes already present in the database, any one of the tested platforms would be adequate and the overall cost of the assay, turn-around-time, and ease of data analysis would be critical factors for consideration. However, if the primary objective is the discovery of novel miRNA transcripts, miRNA-Seq would be the preferred method. Currently, methods for miRNA-Seq-based analyses readily allow for the concurrent multiplexing of up to 48 samples. Together with improved sequencing chemistries and optimized flow cell capacities, miRNA-Seq has become much more cost competitive with array-based technologies. However, the data pre-processing steps, such as de-multiplexing and read mapping remain complex, often requiring substantial informatics and programming support not readily available to individual laboratories. This too is rapidly evolving with the development of off-the-shelf software packages that employ relatively common computing power to obtain differential expression patterns.

## Materials and Methods

### Sample Collection and Processing

Tissue samples were retrieved from sample archives, according to a protocol that was approved by the Mayo Clinic Institutional Review Board with written informed consent, and were de-identified for this work. In order to compare the various miRNA expression profiling platforms, replicates from three types of samples were utilized (a total of six samples); 1) fresh frozen (FF); 2) formalin-fixed paraffin embedded (FFPE) tissue from normal human lung and lung tumors, and 3) lung carcinoma cell lines (Figure 1). Total RNA was extracted in duplicate from one FF tissue sample, designated FF1 and FF2, by using the Qiagen miRNeasy kit (Valencia, CA). Likewise, total RNA from matched FFPE samples were also extracted in duplicate, using the RecoverAll kit (Life Technologies, Grand Island, NY), and identified as FFPE9a and FFPE9b. Therefore, the same human lung tissue was used as the source for both FF and FFPE samples. The FF sample replicates were snap frozen immediately post-surgery. The paraffin samples were kept at RT for approximately two years prior to sectioning and RNA extraction. The human lung cell line, H1299, was cultured as described previously and extracted according to the Qiagen miRNeasy kit protocol [37] and two samples were also used from this sample type, designated H1299-1 and H1299-2.

### Affymetrix miRNA Arrays

Samples were labeled using the Genisphere FlashTag Biotin HSR kit (Hatfield, PA). Briefly, one microgram of total RNA was incubated with ATP and Poly A polymerase to add a 3′ polyA tail. A ligation reaction was then performed to covalently attach to the miRNA population a multiple-biotin molecule containing a 3DNA dendrimer. Labeled samples were subsequently processed according to manufacturer's instructions for the Affymetrix miRNA Array 1.0 (Santa Clara, CA). After hybridization for 16 h at 48°C, the arrays were washed and stained in an Affymetrix Fluidics station 450, then scanned in an Affymetrix 3000 7G scanner.

### Agilent miRNA Arrays

The Human miRNA v2 Microarray Kit (8×15K) was used according to manufacturer's instructions to profile miRNA transcripts on the Agilent Technologies miRNA platform (Santa Clara, CA). Briefly, the Agilent Spike-In control was combined with 100 ng of total RNA sample and both were subjected to dephosphorylation and Cyanine3-pCp ligation. Samples were purified using BioRad MicroBioSpin 6 columns (Hercules, CA) prior to drying and assembly of the hybridization solution. Arrays were hybridized in a 45 µl volume with rotation at 20 rpm for 20 h at 55°C. Agilent Gene Expression Wash Buffers 1(RT) and 2(37°C) were used after hybridization as recommended for the Agilent miRNA Microarray System. Agilent arrays were scanned on a GenePix 4000B scanner (Molecular Devices, Sunnyvale, CA) using 5 µm resolution.

### Illumina miRNA Arrays

Samples were analyzed according to manufacturer's instructions for the now discontinued Illumina miRNA array (San Diego, CA). Briefly, 200 ng of total RNA was reverse transcribed with biotinylated oligo(dT) and random nonamer primers. The resulting cDNA was annealed to chimeric query oligonucleotides, which contain a gene-specific region and a universal primer sequence for PCR amplification, and then bound to streptavidin-conjugated paramagnetic particles. The gene-specific oligonucleotides were extended by second-strand cDNA synthesis and then ligated. Subsequently, the products were sequestered by magnetic separation, washed to remove unbound molecules, and then amplified by PCR with fluorophore-labeled universal primers. The resulting PCR products were purified, applied to HumanRef-8 v3 beadchips (Illumina), and then hybridized for 16 h at 58°C. The beadchips were washed and then scanned in a BeadArray Reader using BeadScan v3 software (Illumina). Quality control parameters were determined to be within normal ranges before proceeding to the final data reduction. Raw, non-normalized, Illumina intensity values were used to compare across platforms.

### NanoString nCounter Analysis

Total RNA samples were analyzed according to manufacturer's instructions for the nCounter Human miRNA Expression Assay kit (NanoString, Seattle, WA). Briefly, 100 ng of each total RNA sample was used as input into the nCounter Human miRNA sample preparation. Hybridization was conducted for 16 h at 65°C. Subsequently, the strip tubes were placed into the nCounter Prep Station for automated sample purification and subsequent reporter capture. Each sample was scanned for 600 FOV on the nCounter Digital Analyzer. Data was extracted using the nCounter RCC Collector.

### Fluidigm Dynamic Array Quantitative PCR

Samples were analyzed by real-time PCR according to the manufacturer's instructions for the Fluidigm dynamic array (South San Francisco, CA). All PCR amplification reagents were purchased from Applied Biosystems, Inc. (Foster City, CA). Briefly, 50 ng of total RNA was reverse transcribed in a 15 µl reaction mixture containing 0.2 µl of 100 nM dNTP, 0.2 µl of RNase inhibitor 20 U/µl, 1.5 µl of reverse transcriptase (50 U/µl), 8 µl of 96-plex reverse primer (Applied Biosystems); mixed to allow a final concentration of 0.05X of each) and 1.6 µl of dH_2_O. Fifty nanograms of total RNA was added to the reaction mixture and incubated as follows; 16°C for 30 min, 42°C for 30 min and then 85°C for 5 min.

Pre-amplification of cDNA was then initiated by creating a pool of 96 TaqMan miRNA Assays at a final concentration of 0.2X for each assay. The pre-PCR amplification reaction was performed in a 10 µl reaction mixture containing 5 µl TaqMan PreAmp Master Mix (2X), 2.5 µl of 96-pooled TaqMan assay mix (0.2X) and 2.5 µl of cDNA. The pre-amplification PCR was performed according to the following cycling conditions: one cycle 95°C for 10 min, 10 cycles at 95°C for 15 sec and then 60°C for 4 min. After pre-amplification PCR, the product was diluted 1:5 with dH_2_O and stored at −80°C until needed for amplification.

Quantitative PCR of the miRNA targets was carried out using the 96.96 dynamic array (Fluidigm Corporation, CA, USA) following manufacturer's protocol. Briefly, a 5 µl sample mixture was prepared for each sample containing 1x TaqMan Universal Master Mix (No UNG), 1X GE Sample Loading Reagent (Fluidigm PN 85000746) and each of diluted pre-amplified cDNA. Five microliters of assay mix were prepared with 1X each of TaqMan miRNA assay and 1X Assay Loading Reagent. The dynamic array was primed with control line fluid in the IFC controller and samples and assay mixes were loaded into the appropriate inlets. The chip was then returned to the IFC controller for loading and mixing, and then placed in the BioMark Instrument for PCR at 95°C for 10 min, followed by 40 cycles at 95°C for 15 sec and 60°C for 1 min. The data was analyzed with Real-Time PCR Analysis Software in the Biomark instrument (Fluidigm Corporation, CA).

### Small RNA Sequencing

One microgram of total RNA sample was treated according to manufacturer's instructions for the Small RNA v1.5 Sample Preparation (Illumina, San Diego, CA). As part of this procedure the small RNA libraries were enriched with 12 cycles of PCR prior to purification on a 6% polyacrylamide gel and excision of the 90–110 bp fraction using GeneCatcher gel tips (San Francisco, CA). The size-selected libraries were run on an Agilent 2100 Bioanalyzer to assess purity and quantitate the miRNA-enriched sample. Samples were diluted and clustered onto single read flow cells using either the Illumina Cluster Station or cBot. Sample containing flow cells were applied to the Illumina GAII_X_ (FF1, FFPE9a, and H1299-1) or HiSeq 2000 (FF2, FFPE9b, and H1299-2; San Diego, CA) instruments for sequencing-by-synthesis using standard Illumina reagents.

### Data Analysis

Data sets were generated by using the least amount of processing allowed by each platform. With the exception of the NGS platform, detected transcripts were defined according to manufacturer criteria for the Affymetrix, Agilent, Illumina, and NanoString platforms respectively. For Figure 3, which provided the fractional deviation from the mean scaled signal, the percent of maximum signal for each platform for each sample was calculated. The mean scaled expression for each miRNA rank was then computed in order to determine the expression decrease across the five platforms, from the top rank down to the bottom rank. Because Illumina is a distinct outlier from the other platforms, the trimmed mean is used for the plot. Next, the deviation from the mean is calculated for each platform, and the fractional deviation was plotted against the mean scaled expression.

#### Affymetrix

Raw data for cross-platform comparisons was extracted without normalization by using the miRNA QC Tool (Affymetrix, Santa Clara, CA). For the purpose of this study, the 847 human miRNA transcripts that are interrogated on Affymetrix miRNA Array 1.0 (miRBase 11.0) were analyzed. Signal intensities with p<0.06 were considered to be detected.

#### Illumina

Data were extracted without background subtraction or normalization in a Sample Probe Profile format by using BeadStudio v3.4 (Illumina). The vendor provided miRNA detection threshold was p<0.05. For this platform, 858 miRNA transcripts were interrogated and available for detection.

#### Agilent

Data was extracted using Agilent Feature Extraction Software v9.5 (Santa Clara, CA). Transcripts detectable by the Agilent platform had a standard error of three times the background. There were 719 miRNAs detectable on this platform.

#### NanoString

Raw data was normalized using internal positive spike controls to account for variability in the hybridization process. The data was further normalized to the average counts of all endogenous miRNAs in each lane to account for any variability in the sample input. MiRNA detection was determined using a metric that yields a detection call at a confidence level of 95% (p<0.05). This detection measure identifies all miRNAs in which the count of the miRNA is two standard deviations above the average of negative spike probes. This platform interrogated 654 miRNA targets.

#### miRNA-Seq

The sequence reads from the Illumina Genome Analyzers were aligned using the Efficient Large-Scale Alignment of Nucleotide Databases (ELAND) algorithm. The Flicker (Illumina) tool was used for processing and initial analysis of miRNA sequencing data including the following steps: 1) trimming the known Illumina adaptor from the reads and exclusion of reads smaller than 15 bp. 2) Alignment of trimmed reads to the genome sequence targets using ELAND for length 15–50 bp. 3) The alignments are sequential in the order mature, iso, loop and then precursor, so a read mapping to mature miRNA is not considered for iso miRNAs. 4). Flicker results were parsed and reported as counts for the miRNA, and these counts were used for expression analysis. Following the primary analysis, counts were scaled by dividing the gene count by the total number of counts for each sample. Then, each data point for each sample was multiplied by the average of the total counts for all lanes. A threshold cutoff of five normalized counts was used as a detected transcript. All counts were then log2 transformed and used in the comparison studies. For purposes of this work, 792 transcripts were considered to be detectable using the miRNA-Seq platform.

#### miRNA PCR

Multivariate analysis was used to pairwise compare miRNA fold-change values across each platform. The miRNA transcript RNU48 was used to normalize qPCR data (MiRNA Ct – RNU48 Ct  =  Δ Ct) and each tissue sample was then calibrated to RNU48-normalized data from the cell line H1299 (Tissue ΔCt – H1299 Δ Ct  =  ΔΔ Ct). Microarray, NanoString and MiRNA-Seq fold-change values represent the difference in miRNA expression between the tissue and the cell line H1299 (log2 Tissue/H1299). Due to the broad range of miRNA expression levels present in these samples, Spearman correlation values are presented.

## Supporting Information

Figure S1 Percent detection among 484 commonly interrogated miRNA transcripts in different sample types. For each sample tested during this study, the percent of miRNA transcripts among those commonly interrogated was plotted.
(TIF)

Figure S2 Pairwise platform comparisons of 484 commonly interrogated miRNA transcripts. The relative agreement of miRNA transcripts that were detected across platforms was assessed in a pair-wise manner by comparing 484 miRNA transcripts that were interrogated within each of the tested platforms.
(TIF)

Table S1 Numerical values for the percent detection among 484 common miRNA transcripts in different sample types.
(DOCX)

Table S2 Numerical values for the commonly detected miRNA transcripts determined from pairwise comparisons of all platforms.
(DOCX)

Table S3 Comparison of Fluidigm-based qPCR with Affymetrix, Agilent, Illumina, Nanostring, and miRNA-Seq platforms. Log transformed data from sample FF1 (Table S3a) and FFPE9a (Table S3b) were compared for 41 and 37, miRNA transcripts, respectively.
(DOCX)

Table S4 Top 50 ranked transcripts determined for each platform. Normalized data were ranked by signal or count for each of the six samples that were tested in this study; A) FF1, B) FF2, C) FFPE9a, D) FFPE9b, E) H1299-1, F) H1299-2.
(PDF)

## References

[1] Bagga S, Bracht J, Hunter S, Massirer K, Holtz J, et al (2005) Regulation by let-7 and lin-4 miRNAsresults in target mRNA degradation. Cell 122: 553–563. 16122423 10.1016/j.cell.2005.07.031

[2] Lee RC, Feinbaum RL, Ambros V (1993) The C. elegans heterochronic gene lin-4 encodes small RNAs with antisense complementarity to lin-14. Cell 75: 843–854. 8252621 10.1016/0092-8674(93)90529-y

[3] Wightman B, Ha I, Ruvkun G (1993) Posttranscriptional regulation of the heterochronic gene lin-14 by lin-4 mediates temporal pattern formation in C. elegans. Cell 75: 855–862. 8252622 10.1016/0092-8674(93)90530-4

[4] Lim LP, Lau NC, Garrett-Engele P, Grimson A, Schelter JM, et al (2005) Microarray analysis shows that some microRNAs downregulate large numbers of target mRNAs. Nature 433: 769–773. 15685193 10.1038/nature03315

[5] Vasudevan S, Tong Y, Steitz JA (2007) Switching from repression to activation: microRNAs can up-regulate translation. Science 318: 1931–1934. 18048652 10.1126/science.1149460

[6] Nottrott S, Simard MJ, Richter JD (2006) Human let-7a miRNA blocks protein production on actively translating polyribosomes. Nature Structural & Molecular Biology 13: 1108–1114. 10.1038/nsmb1173 17128272

[7] Olsen PH, Ambros V (1999) The lin-4 regulatory RNA controls developmental timing in Caenorhabditis elegans by blocking LIN-14 protein synthesis after the initiation of translation. Developmental Biology 216: 671–680. 10642801 10.1006/dbio.1999.9523

[8] Petersen CP, Bordeleau M-E, Pelletier J, Sharp PA (2006) Short RNAs repress translation after initiation in mammalian cells. Molecular Cell 21: 533–542. 16483934 10.1016/j.molcel.2006.01.031

[9] Lau NC, Lim LP, Weinstein EG, Bartel DP (2001) An abundant class of tiny RNAs with probable regulatory roles in Caenorhabditis elegans. Science 294: 858–862. 11679671 10.1126/science.1065062

[10] Rodriguez A, Griffiths-Jones S, Ashurst JL, Bradley A (2004) Identification of mammalian microRNA host genes and transcription units. Genome Research 14: 1902–1910. 15364901 10.1101/gr.2722704 PMC524413

[11] Kozomara A, Griffiths-Jones S (2011) miRBase: integrating microRNA annotation and deep-sequencing data. Nucleic Acids Research 39: D152–157. 21037258 10.1093/nar/gkq1027 PMC3013655

[12] Ambros V (2004) The functions of animal microRNAs. Nature 431: 350–355. 15372042 10.1038/nature02871

[13] Bartel DP (2009) MicroRNAs: target recognition and regulatory functions. Cell 136: 215–233. 19167326 10.1016/j.cell.2009.01.002 PMC3794896

[14] Griffiths-Jones S, Saini HK, van Dongen S, Enright AJ (2008) miRBase: tools for microRNA genomics. Nucleic Acids Research 36: D154–158. 17991681 10.1093/nar/gkm952 PMC2238936

[15] Jiang Q, Wang Y, Hao Y, Juan L, Teng M, et al (2009) miR2Disease: a manually curated database for microRNA deregulation in human disease. Nucleic Acids Research 37: D98–104. 18927107 10.1093/nar/gkn714 PMC2686559

[16] Ambs S, Prueitt RL, Yi M, Hudson RS, Howe TM, et al (2008) Genomic profiling of microRNA and messenger RNA reveals deregulated microRNA expression in prostate cancer. Cancer Research 68: 6162–6170. 18676839 10.1158/0008-5472.CAN-08-0144 PMC2597340

[17] Cohn DE, Fabbri M, Valeri N, Alder H, Ivanov I, et al (2010) Comprehensive miRNA profiling of surgically staged endometrial cancer. American Journal of Obstetrics & Gynecology 202 656: e651–658. 10.1016/j.ajog.2010.02.051 PMC4278076 20400061

[18] Haramati S, Chapnik E, Sztainberg Y, Eilam R, Zwang R, et al (2010) miRNA malfunction causes spinal motor neuron disease. Proceedings of the National Academy of Sciences of the United States of America 107: 13111–13116. 20616011 10.1073/pnas.1006151107 PMC2919953

[19] Iorio MV, Visone R, Di Leva G, Donati V, Petrocca F, et al (2007) MicroRNA signatures in human ovarian cancer. Cancer Research 67: 8699–8707. 17875710 10.1158/0008-5472.CAN-07-1936

[20] Schonrock N, Ke YD, Humphreys D, Staufenbiel M, Ittner LM, et al (2010) Neuronal microRNA deregulation in response to Alzheimer's disease amyloid-beta. PLoS ONE [Electronic Resource] 5: e11070. 10.1371/journal.pone.0011070 PMC2884018 20552018

[21] Thum T, Galuppo P, Wolf C, Fiedler J, Kneitz S, et al (2007) MicroRNAs in the human heart: a clue to fetal gene reprogramming in heart failure.[Erratum appears in Circulation. 2007 Jul 17;116(3): e135]. Circulation 116: 258–267. 17606841 10.1161/CIRCULATIONAHA.107.687947

[22] Landi MT, Zhao Y, Rotunno M, Koshiol J, Liu H, et al (2010) MicroRNA expression differentiates histology and predicts survival of lung cancer. Clinical Cancer Research 16: 430–441. 20068076 10.1158/1078-0432.CCR-09-1736 PMC3163170

[23] van Rooij E, Sutherland LB, Liu N, Williams AH, McAnally J, et al (2006) A signature pattern of stress-responsive microRNAs that can evoke cardiac hypertrophy and heart failure. Proceedings of the National Academy of Sciences of the United States of America 103: 18255–18260. 17108080 10.1073/pnas.0608791103 PMC1838739

[24] Patnaik SK, Kannisto E, Knudsen S, Yendamuri S (2010) Evaluation of microRNA expression profiles that may predict recurrence of localized stage I non-small cell lung cancer after surgical resection. Cancer Research 70: 36–45. 20028859 10.1158/0008-5472.CAN-09-3153

[25] Wang K, Zhang S, Marzolf B, Troisch P, Brightman A, et al (2009) Circulating microRNAs, potential biomarkers for drug-induced liver injury. Proceedings of the National Academy of Sciences of the United States of America 106: 4402–4407. 19246379 10.1073/pnas.0813371106 PMC2657429

[26] Git A, Dvinge H, Salmon-Divon M, Osborne M, Kutter C, et al (2010) Systematic comparison of microarray profiling, real-time PCR, and next-generation sequencing technologies for measuring differential microRNA expression. Rna-A Publication of the Rna Society 16: 991–1006. 10.1261/rna.1947110 PMC2856892 20360395

[27] Li J, Smyth P, Flavin R, Cahill S, Denning K, et al (2007) Comparison of miRNA expression patterns using total RNA extracted from matched samples of formalin-fixed paraffin-embedded (FFPE) cells and snap frozen cells. BMC Biotechnology 7: 36. 17603869 10.1186/1472-6750-7-36 PMC1914054

[28] Pradervand S, Weber J, Lemoine F, Consales F, Paillusson A, et al (2010) Concordance among digital gene expression, microarrays, and qPCR when measuring differential expression of microRNAs. Biotechniques 48: 219–222. 20359303 10.2144/000113367

[29] Sato F, Tsuchiya S, Terasawa K, Tsujimoto G (2009) Intra-platform repeatability and inter-platform comparability of microRNA microarray technology. PLoS ONE [Electronic Resource] 4: e5540. 10.1371/journal.pone.0005540 PMC2677665 19436744

[30] Hasemeier B, Christgen M, Kreipe H, Lehmann U (2008) Reliable microRNA profiling in routinely processed formalin-fixed paraffin-embedded breast cancer specimens using fluorescence labelled bead technology. BMC Biotechnology 8: 90. 19038028 10.1186/1472-6750-8-90 PMC2605753

[31] Zhang X, Chen J, Radcliffe T, Lebrun DP, Tron VA, et al (2008) An array-based analysis of microRNA expression comparing matched frozen and formalin-fixed paraffin-embedded human tissue samples. Journal of Molecular Diagnostics 10: 513–519. 18832457 10.2353/jmoldx.2008.080077 PMC2570634

[32] Geiss GK, Bumgarner RE, Birditt B, Dahl T, Dowidar N, et al (2008) Direct multiplexed measurement of gene expression with color-coded probe pairs. Nature Biotechnology 26: 317–325. 10.1038/nbt1385 18278033

[33] Vaz C, Ahmad HM, Sharma P, Gupta R, Kumar L, et al (2010) Analysis of microRNA transcriptome by deep sequencing of small RNA libraries of peripheral blood. BMC Genomics 11: 288. 20459673 10.1186/1471-2164-11-288 PMC2885365

[34] Gaur A, Jewell DA, Liang Y, Ridzon D, Moore JH, et al (2007) Characterization of microRNA expression levels and their biological correlates in human cancer cell lines. Cancer Research 67: 2456–2468. 17363563 10.1158/0008-5472.CAN-06-2698

[35] Peng Z, Cheng Y, Chin-MingTan B, Kang L, Tian Z, et al (2012) Comprehensive analysis of RNA-Seq data reveals extensive RNA editing in a human transcriptome. Nature Biotechnology 30: 253–262. 10.1038/nbt.2122 22327324

[36] Wang B, Howel P, Bruheim S, Ju J, Owen LB, et al (2011) Systematic evaluation of three microRNA profiling platforms: microarray, beads array, and quantitative real-time PCR array. PLoS ONE [Electronic Resource] 6: e17167. 10.1371/journal.pone.0017167 PMC3037970 21347261

[37] Jang J, Simon V, Feddersen R, Rakhshan F, Schultz D, et al (2011) Quantitative miRNA Expression Analysis Using Fluidigm Microfluidics Dynamic Arrays. BMC Genomics 12: 144. 21388556 10.1186/1471-2164-12-144 PMC3062620

