# Correction: Multi-Platform Analysis of MicroRNA Expression Measurements in RNA from Fresh Frozen and FFPE Tissues

**DOI:** 10.1371/annotation/7d397301-705d-46bb-92cc-ce725975273a

**Published:** 2014-01-08

**Authors:** Christopher P. Kolbert, Rod M. Feddersen, Fariborz Rakhshan, Diane E. Grill, Gyorgy Simon, Sumit Middha, Jin Sung Jang, Vernadette Simon, Debra A. Schultz, Michael Zschunke, Wilma Lingle, Jennifer M. Carr, E. Aubrey Thompson, Ann L. Oberg, Bruce W. Eckloff, Eric D. Wieben, Peter Li, Ping Yang, Jin Jen

There are several errors in the values found in Table 1. A corrected version can be found here: http://plosone.org/corrections/pone.0052517.t001.cn.tif

Following this change, several corrections to the text and figures are necessary.

In the first paragraph of the Results section, the third sentence from the end should read: The miRNA detection count obtained by the NanoString platform ranged from 259 for FFPE9b to 103 for H1299-2 and replicate correlations ranged from 0.950 to 0.967.

In the second paragraph of the Results section, Correlation coefficients “r” should be shown as "R^2^."

In the third paragraph of the Results section, there are two errors.

-The fifth sentence should read: However, detection by Affymetrix was nearly 10% higher in FF2 than FF1.

-The third to last sentence should read: Indeed, both Agilent and NanoString platforms exhibited detection calls only 12% to 20% of the commonly interrogated transcripts in H1299 cells.

In Figures 2A-2D, the Correlation coefficients “r” should be shown as "R^2^."

