# Supplementary figures and images for: Multi-Platform Analysis of MicroRNA Expression Measurements in RNA from Fresh Frozen and FFPE Tissues
Source: PLoS One. 2013 Jan 31;8(1):e52517. doi: 10.1371/journal.pone.0052517 (PMC3561362; doi:10.1371/journal.pone.0052517)

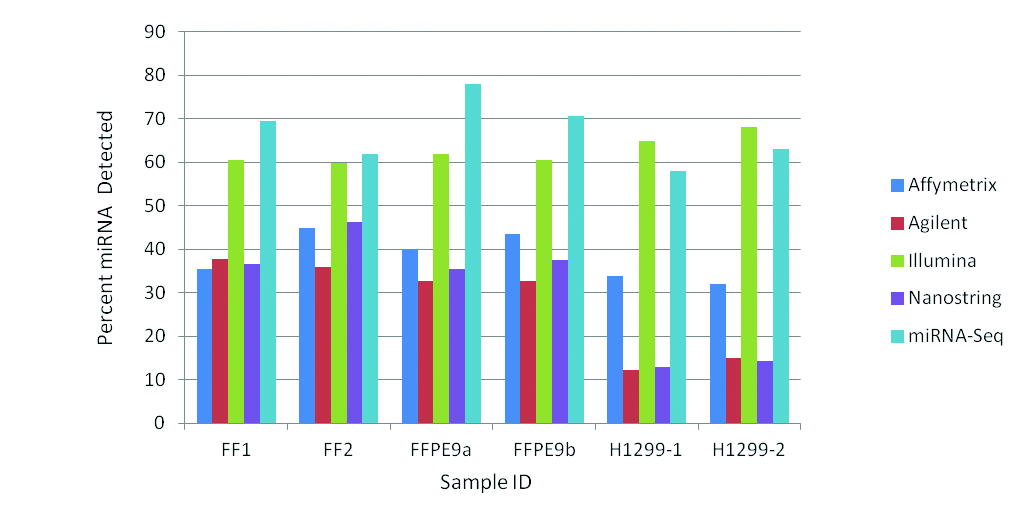

Supplement: Figure S1 — Percent detection among 484 commonly interrogated miRNA transcripts in different sample types. For each sample tested during this study, the percent of miRNA transcripts among those commonly interrogated was plotted. (TIF) [file pone.0052517.s001.tif]

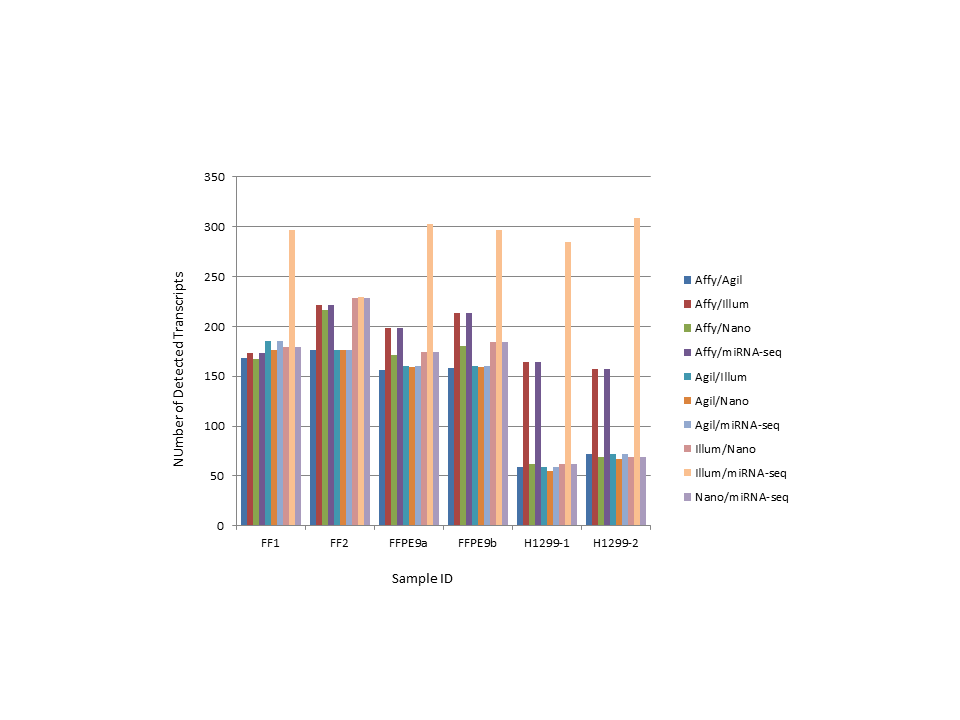

Supplement: Figure S2 — Pairwise platform comparisons of 484 commonly interrogated miRNA transcripts. The relative agreement of miRNA transcripts that were detected across platforms was assessed in a pair-wise manner by comparing 484 miRNA transcripts that were interrogated within each of the tested platforms. (TIF) [file pone.0052517.s002.tif]
