# Supplementary material for: Multi-Platform Analysis of MicroRNA Expression Measurements in RNA from Fresh Frozen and FFPE Tissues
Source: PLoS One. 2013 Jan 31;8(1):e52517. doi: 10.1371/journal.pone.0052517 (PMC3561362; doi:10.1371/journal.pone.0052517)
Supplement: Table S1 — Numerical values for the percent detection among 484 common miRNA transcripts in different sample types. (DOCX) [file pone.0052517.s003.docx]

**Supplemental Table 1. Numerical values for the percent detection among 484 common miRNA transcripts in different sample types.**

|  | Affymetrix | Agilent | Illumina | NanoString | miRNA-Seq |
| --- | --- | --- | --- | --- | --- |
| FF1 | 35.33 | 37.81 | 60.54 | 36.57 | 69.42 |
| FF2 | 44.83 | 35.95 | 59.71 | 46.28 | 61.78 |
| FFPE9a | 40.08 | 32.64 | 61.78 | 35.54 | 78.10 |
| FFPE9b | 43.60 | 32.64 | 60.54 | 37.60 | 70.66 |
| H12991 | 33.88 | 12.19 | 64.88 | 12.81 | 58.06 |
| H12992 | 32.02 | 14.88 | 68.18 | 14.26 | 63.02 |
