# Supplementary material for: Multi-Platform Analysis of MicroRNA Expression Measurements in RNA from Fresh Frozen and FFPE Tissues
Source: PLoS One. 2013 Jan 31;8(1):e52517. doi: 10.1371/journal.pone.0052517 (PMC3561362; doi:10.1371/journal.pone.0052517)
Supplement: Table S2 — Numerical values for the commonly detected miRNA transcripts determined from pairwise comparisons of all platforms. (DOCX) [file pone.0052517.s004.docx]

|  | Affymetrix/  Agilent | Affymetrix/  Illumina | Affymetrix/  Nanostring | Affymetrix/  miRNA-Seq | Agilent/  Illumina | Agilent/  NanoString | Agilent/  miRNA-Seq | Illumina/  NanoString | Illumina/  miRNA-Seq | NanoString/  miRNA-Seq |
| --- | --- | --- | --- | --- | --- | --- | --- | --- | --- | --- |
| FF1 | 168 | 173 | 167 | 173 | 185 | 176 | 185 | 179 | 297 | 179 |
| FF2 | 176 | 221 | 216 | 221 | 176 | 176 | 176 | 228 | 229 | 228 |
| FFPE9a | 156 | 198 | 171 | 198 | 160 | 159 | 160 | 174 | 303 | 174 |
| FFPE9b | 158 | 213 | 180 | 213 | 160 | 159 | 160 | 184 | 297 | 184 |
| H1299-1 | 59 | 164 | 62 | 164 | 59 | 55 | 59 | 62 | 285 | 62 |
| H1299-2 | 72 | 157 | 69 | 157 | 72 | 67 | 72 | 69 | 309 | 69 |

**Supplemental Table 2. Numerical values for the commonly detected transcripts determined from pairwise comparisons of all platforms.**
