# Supplementary material for: Multi-Platform Analysis of MicroRNA Expression Measurements in RNA from Fresh Frozen and FFPE Tissues
Source: PLoS One. 2013 Jan 31;8(1):e52517. doi: 10.1371/journal.pone.0052517 (PMC3561362; doi:10.1371/journal.pone.0052517)
Supplement: Table S3 — Comparison of Fluidigm-based qPCR with Affymetrix, Agilent, Illumina, Nanostring, and miRNA-Seq platforms. Log transformed data from sample FF1 (Table S3a) and FFPE9a (Table S3b) were compared for 41 and 37, miRNA transcripts, respectively. (DOCX) [file pone.0052517.s005.docx]

**Supplemental Table 3a. FF1 comparison of Fluidigm-based qPCR with Affymetrix, Agilent,**

**Illumina, NanoString, and miRNA-Seq.**

| FF1 qPCR | qPCR  log ratio | Affymetrix log ratio | Agilent  log ratio | Illumina  log ratio | Nanostring log ratio | MiRNASeq log ratio |
| --- | --- | --- | --- | --- | --- | --- |
| hsa-miR-125b | -1.137 | 0.664 | 1.685 | -0.328 | 0.470 | -3.153 |
| hsa-miR-92a | 0.464 | -0.839 | 1.497 | -0.636 | 0.259 | -1.468 |
| hsa-miR-221 | -0.607 | -0.224 | 1.296 | -0.839 | 0.714 | -1.162 |
| hsa-let-7a | 1.479 | 3.780 | 3.353 | 0.405 | 4.240 | 0.452 |
| hsa-miR-25 | 0.842 | 2.477 | 2.879 | -0.123 | 2.540 | -1.062 |
| hsa-miR-100 | -1.195 | 0.010 | 1.283 | -0.715 | 0.782 | -2.666 |
| hsa-miR-23a | -0.767 | 1.317 | 3.336 | 0.004 | 3.141 | 0.650 |
| hsa-miR-125a-5p | 0.230 | 0.317 | 2.141 | 0.149 | 1.904 | -1.673 |
| hsa-miR-30d | 2.653 | 2.340 | 3.670 | 0.105 | 4.633 | 0.234 |
| hsa-miR-21 | 3.734 | 8.628 | 7.652 | 2.036 | 8.198 | 0.655 |
| hsa-miR-24 | 0.332 | 2.338 | 4.405 | 0.317 | 3.536 | -1.821 |
| hsa-miR-26a | 2.747 | 3.600 | 6.969 | 0.910 | 5.499 | 1.037 |
| hsa-miR-31 | -0.728 | 0.662 | 3.529 | -1.197 | 2.777 | -0.054 |
| hsa-miR-29a | 1.037 | 6.133 | 6.986 | 1.125 | 6.135 | -0.907 |
| hsa-miR-30c | 1.218 | 3.369 | 6.058 | 0.751 | 5.042 | -2.536 |
| hsa-miR-484 | -0.683 | 0.181 | 1.828 | -0.890 | 2.068 | -0.714 |
| hsa-miR-181a | 3.940 | 0.489 | 4.686 | 0.077 | 3.642 | 2.022 |
| hsa-miR-16 | -0.634 | 4.597 | 6.588 | 1.360 | 8.692 | -0.654 |
| hsa-let-7e | 1.925 | 2.890 | 3.523 | 0.684 | 3.652 | 0.244 |
| hsa-miR-151-5p | 0.954 | 3.227 | 4.292 | 0.897 | 3.767 | 0.599 |
| hsa-miR-27a | -0.090 | 3.765 | 6.821 | 1.463 | 5.585 | -0.710 |
| hsa-miR-20a | 0.581 | 4.853 | 6.898 | 1.224 | 5.670 | -2.735 |
| hsa-miR-425 | -2.498 | 1.880 | 4.035 | 0.330 | 1.986 | -2.662 |
| hsa-miR-455-3p | 0.170 | -0.282 | 1.827 | -1.187 | 0.183 | 1.698 |
| hsa-miR-23b | 6.827 | 3.559 | 7.538 | 1.935 | 5.983 | 4.635 |
| hsa-miR-26b | 1.339 | 3.057 | 8.843 | 2.277 | 5.910 | 2.491 |
| hsa-miR-27b | 5.158 | 7.555 | 8.609 | 2.612 | 8.583 | 3.118 |
| hsa-let-7b | 6.400 | 5.047 | 7.574 | 2.569 | 6.441 | 4.732 |
| hsa-let-7i | 5.603 | 7.073 | 7.299 | 2.821 | 6.701 | 4.591 |
| hsa-miR-126 | 5.176 | 7.810 | 10.266 | 3.223 | 7.721 | 2.583 |
| hsa-let-7f | 1.370 | 8.069 | 4.657 | 3.804 | 4.564 | 2.154 |
| hsa-let-7g | 7.162 | 7.714 | 7.613 | 3.542 | 6.807 | 2.406 |
| hsa-miR-183 | -1.104 | 5.217 | 4.727 | 1.726 | 2.834 | -0.809 |
| hsa-miR-130b | -0.645 | 2.285 | 4.910 | -0.225 | 2.731 | -2.402 |
| hsa-miR-15a | 7.193 | 6.856 | 8.894 | 3.098 | 6.572 | 0.237 |
| hsa-miR-130a | 1.793 | 5.754 | 9.874 | 1.732 | 2.978 | -1.462 |
| hsa-miR-210 | 4.108 | 5.953 | 10.008 | 2.570 | 4.944 | 2.544 |
| hsa-miR-96 | -7.112 | 0.221 | 9.052 | 3.706 | 5.400 | -2.389 |
| hsa-miR-19b | 0.851 | 8.087 | 11.869 | -0.212 | 6.973 | -2.660 |
| hsa-miR-29b | -0.469 | 2.988 | 11.612 | 5.634 | 6.493 | -2.036 |
| hsa-miR-29c | 1.189 | 2.689 | 9.370 | 5.881 | 5.687 | 4.023 |

**Supplemental Table 3b. FFPE comparison of Fluidigm-based qPCR with Affymetrix, Agilent, Illumina, NanoString, and miRNA-Seq.**

| FFPE9a qPCR | qPCR  log ratio | Affymetrix FFPE9a log ratio | Agilent FFPE9a  log ratio | Illumina FFPE9a  log ratio | Nanostring FFPE9a  log ratio | MiRNASeq FFPE9a  log ratio |
| --- | --- | --- | --- | --- | --- | --- |
| hsa-miR-125b | -0.348 | 0.423 | 0.783 | -0.358 | 0.544 | -1.501 |
| hsa-miR-92a | 1.652 | -0.922 | -0.125 | -0.696 | -0.614 | 0.709 |
| hsa-miR-221 | -0.329 | -0.466 | -1.323 | -0.481 | -1.688 | -1.024 |
| hsa-let-7a | -1.136 | 3.505 | 3.081 | 0.363 | 2.868 | -0.101 |
| hsa-miR-25 | 3.612 | 1.632 | 1.521 | -0.173 | 1.141 | 0.046 |
| hsa-miR-100 | 2.368 | 0.273 | 0.656 | -0.451 | 0.074 | -2.694 |
| hsa-miR-125a-5p | -2.688 | -0.733 | 0.696 | 0.114 | 0.284 | 0.071 |
| hsa-miR-30d | 5.882 | 2.361 | 2.531 | 0.166 | 4.322 | 2.267 |
| hsa-miR-21 | 2.298 | 6.551 | 7.350 | 1.926 | 7.064 | 0.013 |
| hsa-miR-24 | 0.446 | 1.918 | 2.410 | 0.256 | 1.851 | -1.714 |
| hsa-miR-26a | 3.215 | 4.027 | 5.265 | 0.958 | 4.053 | 1.861 |
| hsa-miR-31 | -4.349 | -1.767 | -0.564 | -1.907 | -0.157 | -1.146 |
| hsa-miR-29a | 2.718 | 5.419 | 4.705 | 0.823 | 3.721 | 0.064 |
| hsa-miR-30c | 4.463 | 4.034 | 5.282 | 0.843 | 3.392 | -1.255 |
| hsa-miR-484 | -4.667 | 1.373 | 1.152 | -1.935 | 0.985 | 1.362 |
| hsa-miR-181a | 3.111 | 0.403 | 3.403 | 0.352 | 2.417 | 1.366 |
| hsa-miR-16 | -15.450 | 5.057 | 5.267 | 1.472 | 7.437 | 0.941 |
| hsa-let-7e | 0.497 | 1.455 | 2.650 | 0.566 | 2.058 | 0.119 |
| hsa-miR-151-5p | 0.226 | 2.010 | 2.594 | 0.282 | 2.238 | 1.364 |
| hsa-miR-27a | 2.438 | 4.365 | 5.137 | 1.676 | 3.770 | -0.391 |
| hsa-miR-20a | 0.194 | 3.823 | 4.385 | 0.999 | 2.875 | -3.433 |
| hsa-miR-455-3p | -13.419 | -1.763 | 0.043 | -1.773 | 0.317 | 1.970 |
| hsa-miR-23b | 8.174 | 3.395 | 5.983 | 1.787 | 4.521 | 5.758 |
| hsa-miR-26b | -2.827 | 2.606 | 7.994 | 2.176 | 4.224 | 3.303 |
| hsa-miR-27b | 6.602 | 7.336 | 6.888 | 2.642 | 7.526 | 2.637 |
| hsa-let-7b | 5.711 | 4.831 | 7.276 | 2.938 | 5.795 | 4.644 |
| hsa-let-7i | 10.713 | 6.915 | 6.274 | 2.844 | 5.203 | 5.291 |
| hsa-miR-126 | 7.308 | 8.751 | 9.828 | 3.746 | 7.292 | 3.341 |
| hsa-let-7f | -8.004 | 6.718 | 4.080 | 3.568 | 2.695 | 2.024 |
| hsa-let-7g | 6.379 | 7.035 | 6.655 | 3.374 | 5.352 | 2.523 |
| hsa-miR-130b | -1.544 | 1.391 | 2.801 | 0.467 | 1.503 | -2.287 |
| hsa-miR-15a | 4.517 | 5.892 | 6.708 | 3.193 | 4.532 | 2.008 |
| hsa-miR-130a | 3.247 | 5.587 | 7.753 | 2.883 | 2.104 | -0.697 |
| hsa-miR-210 | 2.753 | 5.681 | 7.118 | 3.100 | 2.040 | 2.846 |
| hsa-miR-19b | -1.775 | 5.972 | 8.643 | 2.998 | 4.515 | -1.857 |
| hsa-miR-29b | -8.768 | 2.436 | 7.998 | 5.666 | 2.758 | -2.177 |
| hsa-miR-29c | 2.738 | 2.371 | 7.071 | 5.877 | 3.884 | 5.531 |
