# Supplementary material for: Multi-Platform Analysis of MicroRNA Expression Measurements in RNA from Fresh Frozen and FFPE Tissues
Source: PLoS One. 2013 Jan 31;8(1):e52517. doi: 10.1371/journal.pone.0052517 (PMC3561362; doi:10.1371/journal.pone.0052517)
Supplement: Table S4 — Top 50 ranked transcripts determined for each platform. Normalized data were ranked by signal or count for each of the six samples that were tested in this study; A) FF1, B) FF2, C) FFPE9a, D) FFPE9b, E) H1299-1, F) H1299-2. (PDF) [file pone.0052517.s006.pdf]

A.

Affymetrix FF1 miRNA	Signal	Agilent FF1 miRNA	Signal	Illumina FF1 miRNA	Signal	NanoString FF1 miRNA	Signal	MiRNA-Seq FF1 miRNA	Count
hsa-let-7b	19852.70	hsa-miR-21	65265.30	hsa-miR-21	50924.00	hsa-miR-720	73878.35	hsa-miR-21	2629366.22
hsa-miR-200c	16957.67	hsa-miR-923	46944.50	hsa-let-7f	23817.00	hsa-miR-21	24074.65	hsa-let-7a	1501965.18
hsa-miR-24	12576.29	hsa-miR-451	35302.70	hsa-miR-223	22088.00	hsa-miR-26a	13617.78	hsa-let-7f	1230884.79
hsa-let-7a	11037.70	hsa-miR-22	22525.30	hsa-let-7a	21461.00	hsa-miR-16	11446.75	hsa-miR-143	897713.45
hsa-let-7c	9282.65	hsa-miR-29b	20243.10	hsa-miR-29a	21289.00	hsa-miR-29a	8088.64	hsa-miR-126	306222.93
hsa-let-7d	9101.07	hsa-miR-142-3p	19975.40	hsa-miR-26a	21193.00	hsa-miR-126	6653.11	hsa-let-7b	294545.40
hsa-let-7e	8481.88	hsa-miR-29a	18104.60	hsa-miR-451	20352.00	hsa-miR-27b	5182.09	hsa-miR-148a	253246.62
hsa-miR-103	8221.84	hsa-miR-141	17746.70	hsa-miR-126	20220.00	hsa-let-7a	5122.94	hsa-miR-142-3p	245866.57
hsa-miR-26a	7747.92	hsa-miR-16	13271.20	hsa-let-7g	18983.00	hsa-miR-19b	3565.15	hsa-miR-200c	226172.28
hsa-miR-23b	6076.97	hsa-miR-23a	12892.00	hsa-miR-16	18820.00	hsa-let-7b	3287.12	hsa-let-7g	209599.55
hsa-miR-23a	5943.40	hsa-miR-24	12713.40	hsa-miR-1246	18198.00	hsa-miR-200c	3119.51	hsa-miR-26a	209108.26
hsa-miR-107	5880.19	hsa-let-7a	12333.90	hsa-miR-27a	18118.00	hsa-miR-30b	2809.92	hsa-miR-24	206194.20
hsa-miR-191	5530.13	hsa-miR-27a	12291.40	hsa-miR-148a	17657.00	hsa-miR-451	2790.21	hsa-miR-146b-5p	180602.61
hsa-miR-16	4381.85	hsa-miR-200c	12073.20	hsa-miR-27b	17542.00	hsa-let-7i	2788.23	hsa-miR-27b	169505.05
hsa-miR-151-5p	4292.14	hsa-miR-19b	10192.60	hsa-miR-200c	17478.00	hsa-miR-15a	2699.50	hsa-miR-181a	148996.66
hsa-miR-93	3826.31	hsa-miR-223	9695.05	hsa-miR-26b	16898.00	hsa-miR-24	2614.71	hsa-miR-182	128929.90
hsa-miR-320a	3400.08	hsa-let-7b	9643.67	hsa-miR-125b	16747.00	hsa-miR-20a	2488.51	hsa-miR-451	128268.34
hsa-miR-320b	3333.50	hsa-let-7f	9619.00	hsa-miR-125a-5p	16365.00	hsa-miR-223	2143.43	hsa-miR-10a	106644.34
hsa-miR-221	3205.09	hsa-miR-23b	9024.99	hsa-miR-199a*	16344.00	hsa-let-7g	2117.80	hsa-miR-27a	93446.82
hsa-miR-31	3087.51	hsa-miR-26a	8380.38	hsa-miR-29c	16325.00	hsa-miR-630	2103.99	hsa-miR-29a	84048.40
hsa-miR-125b	3006.30	hsa-miR-15a	8155.44	hsa-let-7b	16078.00	hsa-miR-141	2102.02	hsa-miR-93	75935.85
hsa-let-7i	3005.57	hsa-miR-29c	7928.89	hsa-let-7i	15676.00	hsa-miR-23a	2072.44	hsa-let-7c	66331.69
hsa-miR-320c	2682.47	hsa-miR-15b	7056.88	hsa-miR-1308	15649.00	hsa-miR-103	2056.67	hsa-miR-100	60296.05
hsa-miR-145	2641.15	hsa-miR-27b	6963.79	hsa-miR-150	15598.00	hsa-miR-205	2007.37	hsa-miR-20a	60265.90
hsa-miR-17	2592.93	hsa-miR-34a	6925.70	hsa-miR-24	15144.00	hsa-miR-125b	1914.69	hsa-miR-99b	52032.75
hsa-miR-182	2524.83	hsa-miR-1225-5p	6751.00	hsa-miR-23a	15073.00	hsa-miR-144	1709.62	hsa-miR-146a	46059.19
hsa-miR-92a	2505.67	hsa-let-7i	6456.80	hsa-miR-145	14802.00	hsa-miR-1274a	1705.67	hsa-miR-141	44055.00
hsa-miR-923	2464.13	hsa-miR-199a-3p	6273.63	hsa-miR-23b	14603.00	hsa-miR-142-3p	1683.98	hsa-miR-29b	43604.50
hsa-miR-106a	2451.79	hsa-miR-103	6184.96	hsa-miR-30c	14555.00	hsa-miR-92a	1660.32	hsa-let-7d	41965.67
hsa-miR-1826	2369.58	hsa-miR-126	5884.60	hsa-miR-30d	14348.00	hsa-miR-1274b	1642.57	hsa-miR-422a	40958.25
hsa-miR-222	2196.92	hsa-miR-125b	5471.61	hsa-miR-25	14318.00	hsa-miR-143	1553.84	hsa-miR-151-3p	38602.88
hsa-miR-155	2176.47	hsa-miR-210	5438.41	hsa-miR-29b	14050.00	hsa-let-7d	1463.13	hsa-let-7i	38207.36
hsa-miR-185	2166.94	hsa-miR-200b	5341.56	hsa-miR-374a	13875.00	hsa-miR-199a	1344.82	hsa-let-7e	37233.65
hsa-miR-143	1991.56	hsa-miR-106b	5306.57	hsa-miR-20a	13663.00	hsa-miR-181a	1299.47	hsa-miR-186	36016.94
hsa-miR-20a	1901.86	hsa-miR-20a	5303.65	hsa-miR-142-3p	13553.00	hsa-miR-29b	1277.78	hsa-miR-148b	35016.62
hsa-miR-181a	1870.79	hsa-miR-130a	5145.69	hsa-miR-222	13438.00	hsa-miR-23b	1196.93	hsa-miR-30a	34686.73
hsa-miR-210	1811.16	hsa-let-7g	4758.95	hsa-miR-199a-3p	13381.00	hsa-let-7e	1189.04	hsa-miR-126*	34333.77
hsa-miR-205	1790.54	hsa-miR-107	4450.34	hsa-miR-92a	13283.00	hsa-miR-1260	1173.27	hsa-miR-34c-5p	34165.28
hsa-miR-768-3p	1750.84	hsa-miR-181a	3984.42	hsa-miR-126*	13181.00	hsa-miR-106b	1133.83	hsa-miR-17	34053.54
hsa-miR-126	1745.77	hsa-miR-638	3889.92	hsa-miR-424	13016.00	hsa-miR-96	1131.86	hsa-miR-199a-5p	33911.65
hsa-miR-99b	1700.87	hsa-miR-30d	3743.92	hsa-miR-205	12993.00	hsa-miR-148a	1035.24	hsa-miR-191	33175.60
hsa-miR-29a	1565.98	hsa-let-7e	3497.02	hsa-miR-151-5p	12719.00	hsa-miR-150	1029.32	hsa-miR-183	32863.44
hsa-miR-214	1563.06	hsa-miR-205	3145.42	hsa-miR-1274b	12569.00	hsa-miR-544	1009.60	hsa-miR-30d	32673.66
hsa-miR-342-3p	1441.73	hsa-miR-151-5p	3052.44	hsa-miR-768-5p	11702.00	hsa-miR-221	987.91	hsa-miR-30e	28972.12
hsa-miR-125a-5p	1435.37	hsa-miR-331-3p	2983.33	hsa-miR-15b	11620.00	hsa-miR-320c	922.84	hsa-miR-16	28823.13
hsa-miR-768-5p	1415.74	hsa-let-7d	2979.64	hsa-miR-22	11609.00	hsa-miR-145	905.09	hsa-miR-30c	27544.35
hsa-miR-22	1400.39	hsa-miR-30b	2931.30	hsa-miR-768-3p	11158.00	hsa-miR-200b	871.57	hsa-miR-221	26263.79
hsa-miR-15b	1327.92	hsa-miR-26b	2889.75	hsa-let-7e	11157.00	hsa-let-7f	857.77	hsa-miR-101	26029.68
hsa-miR-181b	1283.30	hsa-miR-17	2878.70	hsa-miR-93	11130.00	hsa-miR-15b	812.41	hsa-miR-22	25944.54
hsa-miR-195	1250.48	hsa-miR-199a-5p	2741.47	hsa-let-7d	11104.00	hsa-miR-26b	812.41	hsa-miR-23a	25350.38

B.

Affymetrix FF2 miRNA	Signal	Agilent FF2 miRNA	Signal	Illumina FF2 miRNA	Signal	Nanostring FF2 miRNA	Signal	MiRNAseq FF2 miRNA	Counts
hsa-let-7b	22,883.35	hsa-miR-21	65,452.80	hsa-miR-21	44,989.00	hsa-miR-720	81,782.21	hsa-miR-21	3,015,999.03
hsa-let-7a	20,584.06	hsa-miR-923	42,479.10	hsa-let-7f	16,304.00	hsa-miR-21	46,196.27	hsa-let-7f	1,260,444.45
hsa-miR-200c	17,136.23	hsa-miR-451	28,689.90	hsa-miR-223	16,006.00	hsa-miR-26a	12,409.11	hsa-let-7a	1,184,178.37
hsa-miR-24	15,480.07	hsa-miR-22	16,605.90	hsa-miR-451	15,466.00	hsa-miR-16	10,700.27	hsa-miR-148a	600,592.95
hsa-let-7c	15,252.88	hsa-miR-29b	15,378.40	hsa-let-7a	15,401.00	hsa-miR-29a	7,477.45	hsa-miR-143	584,388.42
hsa-miR-26a	14,314.68	hsa-miR-142-3p	14,499.10	hsa-miR-126	14,955.00	hsa-miR-126	6,375.69	hsa-miR-126	354,502.48
hsa-let-7d	12,720.29	hsa-miR-29a	13,865.90	hsa-miR-29a	14,846.00	hsa-let-7a	4,989.13	hsa-miR-200c	353,148.35
hsa-let-7e	11,158.22	hsa-miR-141	12,192.10	hsa-miR-26a	14,207.00	hsa-miR-27b	4,876.70	hsa-miR-24	220,617.64
hsa-miR-23a	10,457.71	hsa-let-7a	11,489.30	hsa-miR-27a	13,674.00	hsa-miR-142-3p	4,574.41	hsa-let-7g	180,542.52
hsa-miR-103	10,292.87	hsa-miR-16	10,588.60	hsa-miR-1246	13,279.00	hsa-miR-125b	3,495.14	hsa-miR-26a	179,181.82
hsa-miR-23b	10,105.72	hsa-miR-23a	10,431.40	hsa-miR-27b	13,255.00	hsa-miR-200c	3,495.14	hsa-miR-146b-5p	172,599.26
hsa-miR-107	8,129.97	hsa-miR-24	9,530.13	hsa-let-7g	13,239.00	hsa-miR-19b	3,320.26	hsa-miR-142-3p	154,921.93

hsa-miR-16	7,958.35	hsa-miR-27a	9,052.96	hsa-miR-16	13,228.00	hsa-let-7b	3,307.76	hsa-let-7b	148,944.44
hsa-miR-191	7,238.84	hsa-miR-200c	8,960.49	hsa-miR-148a	13,070.00	hsa-miR-451	3,007.97	hsa-miR-27b	144,641.90
hsa-miR-151-5p	6,469.12	hsa-let-7f	8,778.43	hsa-miR-26b	12,431.00	hsa-miR-24	2,580.76	hsa-miR-148b	132,029.77
hsa-let-7i	6,130.35	hsa-let-7b	8,346.19	hsa-miR-29c	12,343.00	hsa-miR-30b	2,560.77	hsa-miR-10a	125,500.68
hsa-miR-221	5,739.25	hsa-miR-223	7,860.23	hsa-let-7i	12,089.00	hsa-miR-15a	2,523.29	hsa-miR-181a	114,425.15
hsa-miR-125b	5,497.75	hsa-miR-23b	7,125.31	hsa-miR-125b	12,071.00	hsa-let-7i	2,410.87	hsa-miR-182	112,042.40
hsa-miR-1826	5,276.36	hsa-miR-19b	7,035.45	hsa-miR-125a-5p	12,043.00	hsa-miR-144	2,370.90	hsa-miR-29a	104,897.46
hsa-miR-145	5,024.46	hsa-miR-15a	6,065.92	hsa-miR-145	11,779.00	hsa-miR-141	2,253.48	hsa-miR-451	100,200.45
hsa-miR-93	4,737.73	hsa-miR-15b	5,858.68	hsa-miR-200c	11,760.00	hsa-let-7g	2,238.49	hsa-miR-27a	84,431.66
hsa-miR-181a	4,564.90	hsa-miR-29c	5,729.31	hsa-let-7b	11,311.00	hsa-miR-20a	2,228.49	hsa-miR-191	82,883.82
hsa-miR-126	4,310.56	hsa-miR-26a	5,697.65	hsa-miR-24	11,262.00	hsa-miR-205	2,196.02	hsa-miR-93	70,285.77
hsa-miR-92a	4,221.10	hsa-miR-199a-3p	5,380.36	hsa-miR-1308	11,186.00	hsa-miR-103	2,183.52	hsa-miR-99b	67,012.31
hsa-miR-31	4,181.17	hsa-miR-34a	5,355.20	hsa-miR-142-3p	10,890.00	hsa-miR-223	2,118.57	hsa-miR-20a	62,565.77
hsa-miR-222	4,141.38	hsa-let-7i	5,292.66	hsa-miR-199a*	10,793.00	hsa-miR-92a	2,096.08	hsa-miR-16	49,588.26
hsa-miR-125a-5p	4,021.68	hsa-miR-1225-5p	5,216.75	hsa-miR-30c	10,454.00	hsa-miR-23a	1,968.67	hsa-miR-151-3p	48,211.62
hsa-miR-17	3,928.47	hsa-miR-27b	5,211.42	hsa-miR-150	10,369.00	hsa-miR-630	1,906.21	hsa-miR-17	47,436.29
hsa-miR-143	3,697.83	hsa-miR-103	4,577.34	hsa-miR-23a	10,127.00	hsa-miR-143	1,566.44	hsa-let-7e	46,831.22
hsa-miR-923	3,564.53	hsa-miR-126	4,484.57	hsa-miR-29b	10,050.00	hsa-miR-1274a	1,538.96	hsa-miR-146a	46,430.19
hsa-miR-205	3,557.47	hsa-miR-125b	4,331.53	hsa-miR-20a	10,018.00	hsa-miR-1274b	1,449.02	hsa-let-7c	43,729.44
hsa-miR-15b	3,538.37	hsa-miR-200b	4,122.26	hsa-miR-199a-3p	9,965.00	hsa-miR-199a	1,386.56	hsa-miR-125a-5p	42,646.88
hsa-miR-320b	3,440.03	hsa-let-7g	4,088.63	hsa-miR-25	9,844.00	hsa-let-7d	1,381.57	hsa-miR-100	42,515.08
hsa-miR-320a	3,438.78	hsa-miR-210	4,082.41	hsa-miR-222	9,830.00	hsa-miR-150	1,339.09	hsa-miR-30d	40,961.14
hsa-miR-106a	3,320.18	hsa-miR-20a	3,948.72	hsa-miR-126*	9,587.00	hsa-miR-181a	1,234.17	hsa-miR-125b	39,189.09
hsa-miR-182	3,244.07	hsa-miR-106b	3,858.49	hsa-miR-92a	9,481.00	hsa-let-7e	1,189.20	hsa-let-7d	36,724.27
hsa-miR-29a	3,087.32	hsa-miR-130a	3,722.33	hsa-miR-23b	9,398.00	hsa-miR-106b	1,101.76	hsa-miR-183	35,506.63
hsa-miR-320c	3,061.85	hsa-miR-107	3,449.75	hsa-miR-30d	9,280.00	hsa-let-7f	1,094.26	hsa-miR-422a	34,405.78
hsa-miR-155	2,971.13	hsa-miR-638	3,160.34	hsa-miR-205	9,279.00	hsa-miR-1260	1,091.76	hsa-miR-423-5p	34,150.62
hsa-miR-20a	2,956.21	hsa-let-7e	3,157.92	hsa-miR-424	9,089.00	hsa-miR-96	1,089.26	hsa-let-7i	33,238.80
hsa-miR-768-3p	2,940.95	hsa-miR-181a	2,870.01	hsa-miR-22	8,857.00	hsa-miR-221	1,074.27	hsa-miR-126*	32,046.49
hsa-miR-99b	2,620.05	hsa-miR-30d	2,695.10	hsa-miR-374a	8,823.00	hsa-miR-23b	1,064.28	hsa-miR-186	31,049.77
hsa-miR-195	2,579.10	hsa-let-7d	2,652.14	hsa-miR-1274b	8,644.00	hsa-miR-148a	1,036.80	hsa-miR-199a-5p	27,987.85
hsa-miR-22	2,548.32	hsa-miR-151-5p	2,560.18	hsa-miR-93	8,471.00	hsa-miR-320c	974.34	hsa-miR-29b	27,951.74
hsa-miR-210	2,407.32	hsa-miR-331-3p	2,546.93	hsa-miR-151-5p	8,379.00	hsa-miR-29b	944.36	hsa-miR-34c-5p	24,110.27
hsa-miR-768-5p	2,294.17	hsa-miR-26b	2,387.67	hsa-miR-30b	7,898.00	hsa-miR-145	881.90	hsa-miR-210	23,520.21
hsa-miR-185	2,284.71	hsa-miR-205	2,359.24	hsa-miR-15b	7,641.00	hsa-miR-30a	851.92	hsa-miR-22	22,271.61
hsa-miR-342-3p	2,280.81	hsa-miR-30b	2,259.29	hsa-miR-221	7,635.00	hsa-miR-125a-5p	841.93	hsa-miR-30c	22,027.71
hsa-miR-27a	2,255.27	hsa-miR-199a-5p	2,087.12	hsa-miR-768-5p	7,437.00	hsa-miR-200b	839.43	hsa-miR-30a	21,940.47
hsa-miR-199b-3p	2,232.87	hsa-miR-17	2,080.95	hsa-miR-768-3p	7,271.00	hsa-miR-26b	804.46	hsa-miR-23a	20,363.54

C.

Affymetrix FFPE9a miRNA	Signal	Agilent FFPE9a miRNA	Signal	Illumina FFPE9a miRNA	Signal	Nanostring FFPE9a miRNA	Signal	MiRNAseq FFPE9a miRNA	Counts
hsa-let-7b	17091.53	hsa-miR-923	58174.60	hsa-miR-21	47190.00	hsa-miR-720	67359.15	hsa-miR-21	1685815.55
hsa-miR-1826	13959.18	hsa-miR-21	52961.40	hsa-miR-720	44951.00	hsa-miR-21	10973.55	hsa-miR-143	1248821.85
hsa-let-7c	10882.33	hsa-miR-451	20312.10	hsa-miR-1274b	44757.00	hsa-miR-26a	4998.08	hsa-let-7f	1125065.60
hsa-miR-26a	10421.67	hsa-let-7a	10213.70	hsa-miR-1280	32083.00	hsa-miR-126	4942.52	hsa-let-7a	1024149.92
hsa-miR-24	9395.06	hsa-let-7b	7845.31	hsa-miR-126	29056.00	hsa-miR-16	4796.03	hsa-miR-126	517880.02
hsa-let-7a	9124.26	hsa-miR-494	6525.71	hsa-miR-145	24692.00	hsa-miR-27b	2490.20	hsa-miR-26a	370427.80
hsa-miR-200c	8522.07	hsa-let-7f	6446.37	hsa-miR-451	24236.00	hsa-miR-1274a	2113.89	hsa-let-7b	277124.05
hsa-miR-923	7907.77	hsa-miR-638	6274.12	hsa-miR-26a	21911.00	hsa-let-7b	2101.26	hsa-miR-200c	229919.54
hsa-miR-145	7658.29	hsa-miR-23a	5448.76	hsa-miR-27a	21002.00	hsa-miR-125b	2015.40	hsa-let-7g	227382.24
hsa-miR-638	7656.88	hsa-miR-16	5312.51	hsa-miR-1308	20989.00	hsa-let-7a	1980.04	hsa-miR-24	222009.68
hsa-miR-720	6660.94	hsa-miR-1225-5p	5143.56	hsa-let-7a	20845.00	hsa-miR-1260	1805.77	hsa-miR-451	202657.90
hsa-miR-16	6027.66	hsa-miR-22	4814.78	hsa-let-7b	20771.00	hsa-miR-451	1765.36	hsa-miR-10a	195262.22
hsa-miR-103	5738.79	hsa-miR-126	4342.66	hsa-miR-16	20339.00	hsa-miR-1274b	1669.39	hsa-miR-142-3p	175357.80
hsa-miR-23a	5699.81	hsa-miR-27a	3826.00	hsa-let-7f	20221.00	hsa-miR-29a	1517.86	hsa-miR-29a	164776.07
hsa-miR-768-3p	5640.16	hsa-miR-29a	3724.12	hsa-miR-223	19516.00	hsa-miR-142-3p	1118.82	hsa-miR-148a	148970.76
hsa-miR-23b	5423.83	hsa-miR-200c	3500.12	hsa-miR-1246	19130.00	hsa-miR-30b	1101.14	hsa-miR-146b-5p	141051.28
hsa-let-7d	4926.37	hsa-miR-24	3189.47	hsa-miR-143	19118.00	hsa-miR-200c	1085.99	hsa-miR-30d	133670.02
hsa-miR-191	4235.63	hsa-let-7i	3173.21	hsa-miR-27b	17903.00	hsa-miR-23a	1060.73	hsa-miR-27b	121401.56
hsa-miR-107	4117.94	hsa-miR-23b	3071.72	hsa-miR-1260	17534.00	hsa-let-7i	987.49	hsa-miR-27a	116576.83
hsa-miR-149*	3997.65	hsa-miR-223	3035.52	hsa-miR-29a	17261.00	hsa-miR-143	909.20	hsa-miR-146a	105783.65
hsa-miR-320a	3476.81	hsa-miR-125b	2927.41	hsa-miR-1274a	17255.00	hsa-miR-92a	906.68	hsa-miR-30a	105490.52
hsa-miR-126	3352.57	hsa-miR-142-3p	2602.95	hsa-miR-150	16916.00	hsa-miR-150	830.91	hsa-miR-181a	94538.76
hsa-miR-320b	3321.04	hsa-miR-26a	2572.13	hsa-let-7g	16897.00	hsa-miR-24	813.23	hsa-miR-23a	87599.61
hsa-miR-143	3299.11	hsa-let-7g	2449.58	hsa-miR-125b	16402.00	hsa-miR-30a	793.03	hsa-miR-16	87075.81
hsa-miR-1228*	3138.46	hsa-miR-199a-3p	2323.47	hsa-miR-29c	16276.00	hsa-let-7g	772.82	hsa-miR-145	82842.16

hsa-let-7e	3135.71	hsa-miR-34a	2169.60	hsa-miR-125a-5p	15977.00	hsa-miR-630	739.99	hsa-miR-30b	75225.43
hsa-miR-320c	2740.03	hsa-miR-15b	2142.26	hsa-let-7i	15924.00	hsa-miR-320c	661.70	hsa-miR-126*	67094.51
hsa-miR-221	2709.92	hsa-miR-27b	2113.44	hsa-miR-142-3p	15923.00	hsa-miR-15a	656.65	hsa-miR-199a-5p	67022.42
hsa-let-7i	2693.01	hsa-let-7e	1909.91	hsa-miR-26b	15754.00	hsa-miR-19b	649.07	hsa-miR-30c	66935.92
hsa-miR-93	2565.36	hsa-miR-200b	1865.28	hsa-miR-148a	15675.00	hsa-miR-145	636.44	hsa-let-7c	62995.41
hsa-miR-125b	2543.67	hsa-miR-15a	1791.67	hsa-miR-199a-5p	15572.00	hsa-miR-133a	623.81	hsa-let-7i	62043.92
hsa-miR-92a	2364.91	hsa-let-7c	1765.74	hsa-miR-30c	15514.00	hsa-miR-223	611.18	hsa-miR-100	59155.81
hsa-miR-222	2338.99	hsa-miR-30d	1699.92	hsa-miR-200c	15457.00	hsa-miR-103	570.78	hsa-miR-99b	58872.28
hsa-miR-99b	2062.78	hsa-miR-768-3p	1657.92	hsa-miR-30d	14962.00	hsa-miR-181a	555.62	hsa-miR-125a-5p	58540.70
hsa-miR-768-5p	2040.00	hsa-miR-29b	1652.81	hsa-miR-23a	14580.00	hsa-let-7d	550.57	hsa-miR-182	54393.55
hsa-miR-342-3p	2000.50	hsa-miR-181a	1638.08	hsa-miR-24	14515.00	hsa-miR-205	512.69	hsa-miR-191	48747.08
hsa-miR-151-5p	1845.25	hsa-miR-103	1628.37	hsa-miR-29b	14368.00	hsa-miR-544	502.59	hsa-miR-422a	48526.02
hsa-miR-181a	1761.75	hsa-miR-29c	1611.16	hsa-miR-221	14071.00	hsa-miR-199a-3p	497.53	hsa-miR-186	45628.30
hsa-miR-22	1732.84	hsa-let-7d	1605.89	hsa-miR-25	13825.00	hsa-miR-23b	434.40	hsa-miR-125b	42057.81
hsa-miR-205	1695.11	hsa-miR-26b	1603.55	hsa-miR-222	13761.00	hsa-miR-100	411.67	hsa-miR-34c-5p	41673.37
hsa-miR-17	1641.55	hsa-miR-145	1573.27	hsa-miR-30a	13704.00	hsa-let-7e	393.99	hsa-miR-23b	40846.82
hsa-miR-185	1540.54	hsa-miR-30b	1438.23	hsa-miR-126*	13511.00	hsa-miR-200b	363.68	hsa-let-7d	39755.97
hsa-miR-155	1529.14	hsa-miR-30a	1414.41	hsa-miR-23b	13179.00	hsa-miR-20a	358.63	hsa-miR-29b	39534.92
hsa-miR-210	1500.39	hsa-miR-575	1407.12	hsa-miR-768-3p	13141.00	hsa-let-7c	346.00	hsa-miR-93	39323.47
hsa-miR-106a	1408.91	hsa-miR-768-5p	1262.42	hsa-miR-30b	12968.00	hsa-miR-141	320.75	hsa-miR-1260	38468.09
hsa-miR-100	1344.29	hsa-miR-130a	1183.47	hsa-miR-92a	12744.00	hsa-miR-494	313.17	hsa-miR-20a	37136.97
hsa-miR-140-3p	1270.76	hsa-miR-342-3p	1176.65	hsa-miR-199a*	12371.00	hsa-miR-148a	287.91	hsa-miR-22	34863.96
hsa-miR-663	1252.66	hsa-miR-107	1145.85	hsa-miR-199a-3p	12170.00	hsa-miR-222	287.91	hsa-miR-92a	34393.02
hsa-miR-27a	1164.15	hsa-miR-195	1140.38	hsa-miR-594	12095.00	hsa-miR-34a	275.29	hsa-let-7e	34143.14
hsa-miR-214	1126.68	hsa-miR-331-3p	1137.62	hsa-miR-205	11835.00	hsa-miR-590-5p	275.29	hsa-miR-141	32278.60

D.

Affymetrix FFPE9b miRNA	Signal	Agilent FFPE9b miRNA	Signal	Illumina FFPE9b miRNA	Signal	Nanostring FFPE9b miRNA	Signal	MiRNAseq FFPE9b miRNA	Counts
hsa-let-7b	20887.45	hsa-miR-923	49628.40	hsa-miR-1274b	30319.00	hsa-miR-720	73890.67	hsa-let-7a	1294332.41
hsa-let-7a	16631.76	hsa-miR-21	42405.10	hsa-miR-720	24124.00	hsa-miR-126	7141.07	hsa-miR-143	1146467.58
hsa-miR-26a	16439.48	hsa-miR-451	25247.10	hsa-miR-21	22995.00	hsa-miR-26a	7080.40	hsa-miR-21	1052520.68
hsa-let-7c	15083.31	hsa-let-7a	15851.80	hsa-miR-126	18078.00	hsa-miR-16	5019.17	hsa-let-7f	1047867.51
hsa-miR-1826	14797.14	hsa-let-7b	12275.60	hsa-let-7a	17234.00	hsa-miR-21	4635.92	hsa-miR-126	657360.62
hsa-miR-145	13684.13	hsa-let-7f	8470.41	hsa-miR-26a	16703.00	hsa-let-7a	3210.97	hsa-miR-26a	389757.60
hsa-miR-24	12498.17	hsa-miR-23a	7923.31	hsa-miR-451	16415.00	hsa-let-7b	3020.08	hsa-miR-148a	344732.60
hsa-miR-23a	11216.11	hsa-miR-126	6198.01	hsa-miR-223	15712.00	hsa-miR-1274b	2531.78	hsa-let-7b	286857.13
hsa-miR-16	10708.50	hsa-miR-1225-5p	6072.71	hsa-miR-1280	15242.00	hsa-miR-1274a	2488.87	hsa-miR-451	248928.51
hsa-miR-23b	9915.60	hsa-miR-16	6062.18	hsa-miR-145	14978.00	hsa-miR-125b	2459.27	hsa-miR-24	225876.64
hsa-miR-126	9843.08	hsa-miR-638	5602.73	hsa-miR-1308	14249.00	hsa-miR-1260	2102.66	hsa-miR-30d	191735.87
hsa-let-7d	8995.13	hsa-miR-223	5552.55	hsa-miR-125b	14045.00	hsa-miR-451	2033.12	hsa-let-7g	182607.36
hsa-miR-103	8095.62	hsa-miR-22	5114.96	hsa-let-7b	13936.00	hsa-miR-27b	1911.78	hsa-miR-142-3p	181505.88
hsa-miR-200c	7992.36	hsa-miR-494	4904.37	hsa-let-7f	13701.00	hsa-miR-30b	1655.79	hsa-miR-10a	171277.64
hsa-miR-923	7940.57	hsa-miR-24	4383.72	hsa-miR-16	13539.00	hsa-miR-29a	1386.49	hsa-miR-200c	168949.31
hsa-miR-638	6928.65	hsa-miR-125b	3942.99	hsa-miR-1246	13183.00	hsa-miR-23a	1385.01	hsa-miR-29a	132593.90
hsa-miR-768-3p	6588.06	hsa-miR-23b	3916.14	hsa-miR-30c	13147.00	hsa-miR-150	1275.51	hsa-miR-27b	128155.89
hsa-miR-107	6160.62	hsa-miR-29a	3655.98	hsa-miR-1260	13001.00	hsa-miR-223	1168.97	hsa-miR-30a	124408.86
hsa-miR-125b	5995.74	hsa-miR-27a	3626.92	hsa-miR-26b	12981.00	hsa-miR-145	1097.94	hsa-miR-23a	123948.21
hsa-miR-191	5892.69	hsa-miR-142-3p	3510.72	hsa-miR-29a	12834.00	hsa-miR-24	1055.03	hsa-miR-146b-5p	122811.74
hsa-let-7e	5558.66	hsa-let-7g	3235.38	hsa-miR-125a-5p	12773.00	hsa-miR-143	1016.56	hsa-miR-27a	119509.03
hsa-miR-143	5492.96	hsa-miR-26a	3225.54	hsa-miR-150	12771.00	hsa-miR-30a	994.36	hsa-miR-181a	108108.17
hsa-let-7i	4833.94	hsa-miR-15b	3200.11	hsa-miR-30d	12540.00	hsa-let-7i	981.05	hsa-miR-125a-5p	95745.19
hsa-miR-320a	4775.53	hsa-let-7i	3176.76	hsa-let-7g	12432.00	hsa-let-7g	973.65	hsa-miR-145	94865.87
hsa-miR-720	4692.34	hsa-let-7c	2739.51	hsa-miR-27a	12424.00	hsa-miR-630	911.50	hsa-miR-126*	93246.59
hsa-miR-320b	4546.59	hsa-miR-200c	2597.93	hsa-miR-1274a	12175.00	hsa-miR-142-3p	901.14	hsa-miR-125b	91051.78
hsa-miR-221	4522.37	hsa-miR-34a	2542.27	hsa-miR-24	11274.00	hsa-miR-92a	833.08	hsa-miR-16	90079.75
hsa-miR-222	4163.67	hsa-miR-199a-3p	2430.41	hsa-let-7i	11159.00	hsa-miR-103	781.29	hsa-miR-422a	83432.35
hsa-miR-92a	4054.58	hsa-let-7e	2329.43	hsa-miR-23a	11104.00	hsa-miR-200c	776.85	hsa-let-7c	82259.15
hsa-miR-342-3p	3977.15	hsa-miR-30d	2267.74	hsa-miR-148a	11076.00	hsa-miR-181a	677.71	hsa-miR-148b	82196.75
hsa-miR-320c	3953.48	hsa-miR-145	2200.05	hsa-miR-126*	10955.00	hsa-miR-320c	668.83	hsa-miR-146a	77463.69
hsa-miR-181a	3855.90	hsa-let-7d	2142.53	hsa-miR-27b	10375.00	hsa-let-7d	659.95	hsa-miR-191	76961.64
hsa-miR-150	3676.55	hsa-miR-27b	2098.64	hsa-miR-30b	10128.00	hsa-miR-133a	637.75	hsa-miR-30b	74905.03
hsa-miR-195	3473.22	hsa-miR-30b	2090.28	hsa-miR-29c	9834.00	hsa-let-7c	581.53	hsa-miR-30c	74782.58
hsa-miR-151-5p	3470.23	hsa-miR-26b	1950.68	hsa-miR-23b	9503.00	hsa-miR-15a	577.09	hsa-miR-100	71790.09
hsa-miR-768-5p	3428.61	hsa-miR-30a	1929.48	hsa-miR-92a	9162.00	hsa-miR-23b	541.57	hsa-miR-99b	68558.52
hsa-miR-93	3225.47	hsa-miR-768-3p	1928.24	hsa-miR-768-3p	9159.00	hsa-miR-544	541.57	hsa-miR-23b	57377.49
hsa-miR-100	3185.93	hsa-miR-103	1925.45	hsa-miR-25	9083.00	hsa-miR-199a-3p	488.30	hsa-miR-199a-5p	52783.21

hsa-miR-27a	3105.90	hsa-miR-15a	1779.19	hsa-miR-30a	9047.00	hsa-miR-19b	479.43	hsa-let-7d	43963.75
hsa-miR-155	3091.37	hsa-miR-181a	1714.38	hsa-miR-200c	8947.00	hsa-miR-100	477.95	hsa-let-7i	42037.18
hsa-miR-22	2784.97	hsa-miR-195	1698.25	hsa-miR-199a-3p	8105.00	hsa-let-7e	451.31	hsa-miR-186	41829.01
hsa-miR-99b	2628.57	hsa-miR-575	1546.39	hsa-miR-221	7584.00	hsa-miR-20a	424.68	hsa-miR-92a	38303.56
hsa-miR-17	2570.24	hsa-miR-130a	1521.98	hsa-miR-195	7541.00	hsa-miR-30d	390.64	hsa-miR-22	36713.43
hsa-miR-149*	2555.87	hsa-miR-150	1512.55	hsa-miR-22	7513.00	hsa-miR-99a	387.68	hsa-miR-150	36260.36
hsa-miR-30a	2312.80	hsa-miR-107	1498.76	hsa-miR-100	7309.00	hsa-miR-30c	325.54	hsa-miR-30a*	33541.93
hsa-miR-140-3p	2260.87	hsa-miR-342-3p	1491.41	hsa-miR-222	7305.00	hsa-miR-26b	316.66	hsa-miR-93	33400.81
hsa-miR-29a	2253.52	hsa-miR-29c	1480.56	hsa-miR-29b	7223.00	hsa-miR-25	306.30	hsa-miR-1260	33104.01
hsa-miR-106a	2244.01	hsa-miR-30c	1477.52	hsa-miR-142-3p	6929.00	hsa-let-7f	301.86	hsa-let-7e	33000.80
hsa-miR-30c	2159.00	hsa-miR-331-3p	1207.49	hsa-let-7e	6896.00	hsa-miR-200b	300.38	hsa-miR-34c-5p	32495.25
hsa-miR-181b	2144.97	hsa-miR-200b	1184.47	hsa-let-7c	6573.00	hsa-miR-15b	295.94	hsa-miR-423-5p	31903.40

E. Affymetrix H1299-1 miRNA	Signal	Agilent H1299-1 miRNA	Signal	Illumina H1299-1 miRNA	Signal	Nanostring H1299-1 miRNA	Signal	MiRNAseq H1299-1 miRNA	Counts
hsa-miR-1826	7486.11	hsa-miR-923	1987.06	hsa-miR-222	21351.00	hsa-miR-92a	1387.46	hsa-miR-21	1670215.24
hsa-miR-92a	4482.23	hsa-miR-125b	1701.18	hsa-miR-125b	21026.00	hsa-miR-125b	1382.73	hsa-miR-10a	1332240.79
hsa-miR-222	3902.01	hsa-miR-15b	1576.39	hsa-miR-92a	20649.00	hsa-miR-720	990.14	hsa-let-7a	1098333.96
hsa-miR-221	3743.05	hsa-miR-23a	1276.81	hsa-miR-221	19643.00	hsa-miR-221	602.28	hsa-miR-93	839153.77
hsa-miR-923	3444.31	hsa-let-7a	1207.12	hsa-miR-720	17069.00	hsa-miR-100	391.01	hsa-miR-24	728473.40
hsa-miR-24	2486.67	hsa-miR-92a	839.50	hsa-miR-1280	16926.00	hsa-miR-26a	301.14	hsa-miR-182	538968.56
hsa-miR-103	2401.97	hsa-miR-331-3p	815.26	hsa-let-7a	16208.00	hsa-let-7a	271.18	hsa-miR-20a	401203.13
hsa-miR-23a	2385.37	hsa-miR-221	651.10	hsa-miR-1274a	16130.00	hsa-miR-1260	247.53	hsa-miR-100	382768.98
hsa-miR-320a	2134.97	hsa-miR-24	600.20	hsa-miR-25	15587.00	hsa-miR-103	238.07	hsa-miR-191	351079.16
hsa-miR-320b	1978.24	hsa-miR-100	545.77	hsa-miR-100	15493.00	hsa-miR-1274a	236.50	hsa-let-7f	276617.89
hsa-miR-31	1951.00	hsa-let-7f	381.18	hsa-miR-1274b	15088.00	hsa-miR-23a	234.92	hsa-miR-422a	248279.73
hsa-miR-191	1937.53	hsa-miR-103	358.73	hsa-miR-23a	15034.00	hsa-miR-24	225.46	hsa-miR-17	201160.18
hsa-miR-125b	1897.65	hsa-miR-21	324.56	hsa-miR-125a-5p	14758.00	hsa-miR-125a-5p	197.08	hsa-miR-148b	184522.64
hsa-miR-93	1884.54	hsa-miR-125a-5p	310.59	hsa-miR-93	13443.00	hsa-miR-15b	146.63	hsa-miR-29b	178800.66
hsa-miR-320c	1640.59	hsa-miR-25	305.29	hsa-miR-30d	13340.00	hsa-miR-331-3p	132.44	hsa-miR-137	171448.14
hsa-miR-107	1552.09	hsa-let-7e	304.27	hsa-miR-1260	12693.00	hsa-miR-25	121.40	hsa-miR-30c	159732.41
hsa-miR-181a	1332.57	hsa-miR-30d	294.16	hsa-miR-15b	12671.00	hsa-miR-29a	115.10	hsa-miR-29a	157573.46
hsa-miR-125a-5p	1152.06	hsa-miR-93	251.79	hsa-miR-21	12421.00	hsa-miR-181a	104.06	hsa-miR-27a	152878.11
hsa-let-7e	1144.10	hsa-miR-342-3p	236.27	hsa-miR-24	12156.00	hsa-miR-296-5p	97.75	hsa-miR-125b	119059.53
hsa-miR-100	1112.82	hsa-miR-107	232.59	hsa-miR-26a	11278.00	hsa-let-7e	94.60	hsa-miR-26a	101938.87
hsa-miR-638	1038.29	hsa-miR-324-3p	220.10	hsa-miR-31	11259.00	hsa-miR-93	89.87	hsa-miR-99b	75910.64
hsa-let-7a	803.41	hsa-miR-22	208.73	hsa-miR-1246	10956.00	hsa-miR-455-3p	85.14	hsa-miR-425	73495.03
hsa-miR-424*	797.86	hsa-miR-151-5p	155.81	hsa-miR-768-3p	10752.00	hsa-miR-92b	83.56	hsa-miR-96	62972.01
hsa-miR-1308	760.08	hsa-miR-320a	155.39	hsa-miR-544	10673.00	hsa-miR-21	81.99	hsa-miR-18a	62609.67
hsa-miR-1307	739.29	hsa-miR-181a	154.84	hsa-miR-423-3p	10036.00	hsa-miR-125a-3p	66.22	hsa-miR-9	62534.18
hsa-miR-768-5p	709.18	hsa-miR-29a	142.81	hsa-miR-193a-5p	10000.00	hsa-miR-132	66.22	hsa-miR-151-3p	59107.03
hsa-miR-26a	639.15	hsa-miR-16	137.92	hsa-miR-29a	9758.00	hsa-miR-708	64.64	hsa-miR-221	58759.79
hsa-miR-361-5p	617.37	hsa-miR-197	137.21	hsa-miR-320d	9658.00	hsa-miR-423-3p	59.91	hsa-miR-183	57582.18
hsa-miR-423-3p	604.67	hsa-miR-193a-5p	128.73	hsa-miR-768-5p	9194.00	hsa-miR-655	58.34	hsa-miR-125a-5p	55740.27
hsa-let-7b	600.49	hsa-miR-455-3p	114.02	hsa-miR-191	8926.00	hsa-miR-744	58.34	hsa-miR-126	51090.22
hsa-miR-106b*	560.28	hsa-miR-27a	108.75	hsa-miR-92b	8830.00	hsa-miR-423-5p	55.18	hsa-miR-16	45353.13
hsa-miR-17	530.06	hsa-miR-361-5p	102.05	hsa-miR-30c	8650.00	hsa-miR-216a	53.61	hsa-miR-30a	45111.57
hsa-miR-25*	530.06	hsa-miR-768-3p	98.88	hsa-miR-484	8599.00	hsa-miR-193b	52.03	hsa-miR-374a*	44643.55
hsa-miR-99b	522.76	hsa-miR-365	96.22	hsa-miR-331-3p	8437.00	hsa-miR-383	52.03	hsa-miR-19b	43194.18
hsa-miR-23b	515.56	hsa-miR-149	95.37	hsa-miR-181a	8406.00	hsa-miR-20a	48.88	hsa-miR-30a*	40355.84
hsa-miR-342-3p	515.56	hsa-miR-940	94.85	hsa-miR-1201	7907.00	hsa-miR-1297	48.88	hsa-let-7g	39555.66
hsa-miR-574-3p	515.56	hsa-miR-1225-5p	90.62	hsa-miR-1308	7494.00	hsa-miR-99b	44.15	hsa-miR-34c-5p	38951.76
hsa-miR-1180	512.00	hsa-miR-324-5p	90.03	hsa-miR-16	7333.00	hsa-let-7c	39.42	hsa-miR-186	37864.73
hsa-miR-193a-5p	487.75	hsa-miR-193b	86.45	hsa-let-7e	6944.00	hsa-miR-31	39.42	hsa-miR-148a	37260.83
hsa-miR-151-5p	458.25	hsa-miR-106b	79.49	hsa-miR-151-5p	6829.00	hsa-miR-34b	39.42	hsa-miR-181a	36687.12
hsa-miR-106a	418.77	hsa-miR-1228	74.87	hsa-miR-324-3p	6688.00	hsa-miR-548a-5p	39.42	hsa-miR-106b	35751.07
hsa-miR-455-3p	401.71	hsa-miR-17	70.31	hsa-miR-27a	6572.00	hsa-let-7b	37.84	hsa-miR-423-5p	32474.90
hsa-miR-193b	372.22	hsa-miR-26a	66.90	hsa-miR-378	6515.00	hsa-miR-328	37.84	hsa-miR-10b	32248.43
hsa-miR-15b	367.09	hsa-miR-31	66.51	hsa-miR-342-3p	6395.00	hsa-miR-660	37.84	hsa-let-7e	31433.16
hsa-miR-181b	330.84	hsa-miR-1234	65.45	hsa-miR-193b	6098.00	hsa-let-7f	36.26	hsa-miR-30d	27779.55
hsa-miR-149*	328.56	hsa-miR-638	61.33	hsa-miR-20a	5851.00	hsa-miR-518f	36.26	hsa-miR-19a	25379.03
hsa-miR-339-5p	328.56	hsa-miR-30a	61.12	hsa-miR-30a	5418.00	hsa-miR-523	36.26	hsa-miR-301a	23114.40
hsa-miR-378	317.37	hsa-miR-29b-1*	55.14	hsa-miR-615-3p	5314.00	hsa-miR-574-3p	36.26	hsa-miR-378	22993.62
hsa-miR-92b	308.69	hsa-miR-21*	54.45	hsa-miR-296-5p	5238.00	hsa-miR-10a	34.69	hsa-miR-130a	22133.05
hsa-miR-149	306.55	hsa-miR-222	51.10	hsa-miR-425	5204.00	hsa-miR-191	34.69	hsa-miR-92a	21046.03

F.

Affymetrix H1299-2 miRNA	Signal	Agilent H1299-2 miRNA	Signal	Illumina H1299-2 miRNA	Signal	Nanostring H1299-2 miRNA	Signal	MiRNAseq H1299-2 miRNA	Counts
hsa-miR-1826	8364.13	hsa-miR-923	2685.76	hsa-miR-125b	31492.00	hsa-miR-92a	1571.20	hsa-miR-21	2278256.61
hsa-miR-92a	5220.60	hsa-miR-125b	2014.64	hsa-miR-92a	29551.00	hsa-miR-27b	1482.44	hsa-let-7a	975094.56
hsa-miR-221	4389.98	hsa-miR-15b	1709.32	hsa-miR-222	29470.00	hsa-miR-125b	1240.17	hsa-miR-24	846717.97
hsa-miR-222	4211.15	hsa-miR-23a	1480.04	hsa-miR-221	23429.00	hsa-miR-720	1098.64	hsa-miR-10a	824046.23
hsa-miR-923	3983.99	hsa-let-7a	1400.08	hsa-miR-720	22180.00	hsa-miR-221	770.01	hsa-miR-100	649180.08
hsa-miR-23a	2817.11	hsa-miR-92a	987.98	hsa-miR-1274b	21683.00	hsa-miR-544	742.42	hsa-miR-148b	454884.27
hsa-miR-103	2797.65	hsa-miR-331-3p	932.03	hsa-let-7a	20666.00	hsa-miR-133a	609.29	hsa-miR-93	409327.85
hsa-miR-24	2683.69	hsa-miR-221	818.09	hsa-miR-1274a	20641.00	hsa-miR-320c	546.92	hsa-miR-27a	402782.85
hsa-miR-31	2304.12	hsa-miR-24	675.28	hsa-miR-1280	20211.00	hsa-miR-100	520.53	hsa-miR-422a	385441.57
hsa-miR-320a	2304.12	hsa-miR-100	604.45	hsa-miR-100	20128.00	hsa-let-7a	407.79	hsa-miR-191	310785.54
hsa-miR-320b	2256.70	hsa-let-7f	458.62	hsa-miR-23a	19593.00	hsa-miR-26a	381.41	hsa-miR-125b	223858.58
hsa-miR-125b	2241.11	hsa-miR-103	425.76	hsa-miR-93	19026.00	hsa-miR-23a	307.04	hsa-let-7f	200764.00
hsa-miR-191	2179.83	hsa-miR-30d	363.03	hsa-miR-25	18785.00	hsa-miR-1274a	285.45	hsa-miR-151-3p	180934.31
hsa-miR-93	2179.83	hsa-miR-21	347.01	hsa-miR-125a-5p	18621.00	hsa-miR-1260	255.47	hsa-miR-182	177199.42
hsa-miR-107	1686.71	hsa-miR-125a-5p	343.87	hsa-miR-15b	17986.00	hsa-miR-24	250.67	hsa-miR-20a	172888.81
hsa-miR-320c	1663.49	hsa-let-7e	335.30	hsa-miR-30d	17876.00	hsa-miR-16	245.87	hsa-miR-29a	172623.39
hsa-miR-181a	1573.76	hsa-miR-25	331.80	hsa-miR-21	17361.00	hsa-miR-590-5p	238.68	hsa-miR-137	125819.73
hsa-miR-125a-5p	1499.22	hsa-miR-93	306.51	hsa-miR-1260	16618.00	hsa-miR-125a-5p	236.28	hsa-miR-17	121901.55
hsa-miR-100	1314.23	hsa-miR-342-3p	275.36	hsa-miR-24	16407.00	hsa-miR-103	224.29	hsa-miR-26a	114564.10
hsa-let-7e	1260.69	hsa-miR-107	263.27	hsa-miR-768-3p	15801.00	hsa-miR-15b	178.71	hsa-miR-99b	88660.93
hsa-miR-424*	982.29	hsa-miR-324-3p	256.19	hsa-miR-31	15635.00	hsa-miR-25	164.32	hsa-miR-30c	88307.29
hsa-miR-1308	975.50	hsa-miR-22	217.20	hsa-miR-544	15453.00	hsa-miR-29a	140.33	hsa-miR-30a*	74441.57
hsa-miR-638	935.76	hsa-miR-181a	181.28	hsa-miR-193a-5p	15172.00	hsa-miR-296-5p	136.73	hsa-miR-106b	70190.28
hsa-miR-1307	867.07	hsa-miR-151-5p	179.27	hsa-miR-1246	15018.00	hsa-miR-331-3p	125.94	hsa-miR-378	68713.36
hsa-miR-768-5p	843.36	hsa-miR-320a	167.92	hsa-miR-768-5p	14858.00	hsa-miR-125a-3p	124.74	hsa-miR-148a	67131.49
hsa-let-7a	786.88	hsa-miR-29a	163.29	hsa-miR-26a	14838.00	hsa-miR-181a	118.74	hsa-miR-29b	66871.39
hsa-miR-26a	770.69	hsa-miR-193a-5p	147.89	hsa-miR-191	14558.00	hsa-miR-494	118.74	hsa-miR-9	64338.11
hsa-miR-361-5p	754.83	hsa-miR-197	136.56	hsa-miR-320d	14399.00	hsa-miR-93	101.95	hsa-miR-126	59625.20
hsa-miR-423-3p	685.02	hsa-miR-16	131.72	hsa-miR-30c	13998.00	hsa-miR-579	101.95	hsa-miR-425	54992.90
hsa-miR-342-3p	648.07	hsa-miR-1225-5p	128.46	hsa-miR-423-3p	13688.00	hsa-miR-485-3p	92.35	hsa-miR-30a	54566.25
hsa-let-7b	634.73	hsa-miR-149	124.95	hsa-miR-92b	13209.00	hsa-miR-92b	86.36	hsa-miR-183	54008.79
hsa-miR-106b*	600.49	hsa-miR-455-3p	123.62	hsa-miR-331-3p	13098.00	hsa-let-7e	85.16	hsa-miR-221	53897.75
hsa-miR-17	600.49	hsa-miR-365	117.91	hsa-miR-29a	13061.00	hsa-miR-192	83.96	hsa-miR-27b	49835.08
hsa-miR-151-5p	588.13	hsa-miR-768-3p	116.73	hsa-miR-1201	12949.00	hsa-miR-216a	77.96	hsa-miR-374a*	46380.06
hsa-miR-193a-5p	580.04	hsa-miR-27a	110.04	hsa-miR-181a	12827.00	hsa-miR-132	76.76	hsa-miR-23a	39148.31
hsa-miR-25*	580.04	hsa-miR-193b	108.91	hsa-miR-484	11805.00	hsa-miR-455-3p	71.96	hsa-miR-423-5p	38517.84
hsa-miR-23b	576.03	hsa-miR-361-5p	105.77	hsa-miR-1308	11654.00	hsa-miR-20a	67.17	hsa-miR-16	38269.15
hsa-miR-1180	568.10	hsa-miR-940	105.69	hsa-miR-151-5p	11527.00	hsa-miR-423-3p	67.17	hsa-miR-96	35253.71
hsa-miR-99b	568.10	hsa-miR-324-5p	105.19	hsa-miR-16	11253.00	hsa-miR-744	67.17	hsa-miR-25	33972.24
hsa-miR-574-3p	556.41	hsa-miR-106b	103.42	hsa-let-7e	11153.00	hsa-miR-196a	63.57	hsa-miR-30d	33851.32
hsa-miR-106a	501.46	hsa-miR-26a	103.00	hsa-miR-27a	10940.00	hsa-miR-99b	57.57	hsa-miR-125a-5p	33722.79
hsa-miR-455-3p	501.46	hsa-miR-17	81.89	hsa-miR-342-3p	10810.00	hsa-miR-222	57.57	hsa-miR-186	31845.84
hsa-miR-181b	407.31	hsa-miR-31	77.70	hsa-miR-324-3p	10603.00	hsa-miR-1283	57.57	hsa-miR-34c-5p	29720.96
hsa-miR-193b	396.18	hsa-miR-1234	73.07	hsa-miR-193b	9872.00	hsa-let-7i	56.37	hsa-let-7e	28282.82
hsa-miR-339-5p	372.22	hsa-miR-30a	69.17	hsa-miR-30a	9630.00	hsa-miR-574-3p	56.37	hsa-let-7g	26277.34
hsa-miR-149	362.04	hsa-miR-1228	68.89	hsa-miR-20a	9579.00	hsa-miR-708	56.37	hsa-miR-181a	24496.97
hsa-miR-15b	354.59	hsa-miR-23b	68.31	hsa-miR-197	9356.00	hsa-let-7b	53.97	hsa-miR-92a	23913.66
hsa-miR-92b	328.56	hsa-let-7b	67.15	hsa-miR-378	9059.00	hsa-miR-1979	53.97	hsa-miR-130a	22304.41
hsa-miR-149*	321.80	hsa-miR-222	66.86	hsa-miR-425	8748.00	hsa-miR-193b	52.77	hsa-miR-30e*	20893.65
hsa-miR-320d	313.00	hsa-miR-21*	65.39	hsa-miR-574-3p	8491.00	hsa-miR-423-5p	52.77	hsa-miR-151-5p	20168.12
